# Investigation of theoretical scaling laws using large eddy simulations for airborne spreading of viral contagion from sneezing and coughing

**DOI:** 10.1063/5.0054651

**Published:** 2021-06-29

**Authors:** K. Liu, M. Allahyari, J. Salinas, N. Zgheib, S. Balachandar

**Affiliations:** 1 Department of Mechanical and Aerospace Engineering, University of Florida, Gainesville, Florida 32611, USA; 2 School of Engineering, Lebanese American University, Byblos, Lebanon

## Abstract

Using a set of large eddy point-particle simulations, we explore the fluid dynamics of an ejected puff resulting from a cough/sneeze. The ejection contains over 61 000 potentially virus-laden droplets at an injection Reynolds number of about 46 000, comparable to an actual cough/sneeze. We observe that global puff properties, such as centroid, puff volume, momentum, and buoyancy vary little across realizations. Other properties, such as maximum extent, shape, and edge velocity of the puff, may exhibit substantial variation. In many realizations, a portion of the puff splits off and advances along a random direction, while keeping airborne droplet nuclei afloat. This peeled-off portion provides a mechanism for virus-laden droplets to travel over large distances in a short amount of time. We also observe that the vast majority of droplets remain suspended within the puff after all liquid has evaporated. The main objectives of the study are to (i) evaluate assumptions of Balachandar's *et al.* theory [Int. J. Multiphase Flow **132**, 103439 (2020)], which include buoyancy effects, shape of the puff, and droplet evaporation rate, (ii) obtain values of closure parameters, which include location and time of the virtual origin, and puff entrainment and drag coefficients, and (iii) evaluate the accuracy of the theory in predicting the shape, size, and location of the puff, as well as droplet number density long after ejection. The theory adequately predicts global puff properties including size, velocity, and distance traveled, the largest size of droplets that exit the puff due to settling, and the droplet size distribution within the puff long after ejection.

## I. INTRODUCTION

To date, the COVID-19 pandemic has resulted in the loss of millions of lives around the globe and has resulted in the closure of many industries to help slow down the spread of the virus. There are two primary routes by which the virus spreads from an infected person through an expiratory event, such as breathing, talking, coughing, or sneezing. In the direct route, the virus-laden droplets ejected by the infected host fall and deposit directly on the receiving host or on surfaces to be later picked up by the receiving host coming into contact with contaminated surfaces. In the indirect airborne route, some of the ejected droplets that rapidly evaporate remain afloat in the air for longer periods of time and travel toward a receiving host who happens to inhale the evaporated droplet nuclei to become infected with the virus.

Airborne transmission is a complex fluid mechanical problem^2–6^ that is controlled by the trajectories of the virus-laden droplets initially contained within the ejected puff of air. The puff, a finite volume of relatively hot and humid air, is usually ejected alongside thousands of droplets. The motion of the droplets and their trajectories depend primarily on their size. Relatively large droplets travel near-ballistically with trajectories that are minimally affected by the puff.^1^ On the other hand, relatively small droplets follow closely the motion of the puff and are strongly affected by the turbulent vortical structures within. Furthermore, because of the large density difference between the droplets and the surrounding air, all the droplets fall at their terminal velocity with respect to the surrounding air, but the larger droplets fall faster and settle out of the puff. However, the size of droplets continuously decreases due to evaporation until most of the water content has evaporated and the droplet reduces mostly to the nonvolatile droplet nuclei.^1,7–10^ While the fall velocity of a droplet depends only on its size, the rate of evaporation depends on a variety of parameters including the size of the droplet, the nonvolatile composition, and the temperature and humidity of the puff and ambient.

The dynamics of the puff and the droplets within are influenced by a number of parameters, such as ejection volume, ejection velocity, number and size distribution of droplets, ambient conditions, and so on. Furthermore, even nearly identical puffs, with the same nominal value of these parameters, can show considerable variation due to the turbulent nature of the flow, which magnifies small initial variations in the temperature or velocity fields of the puffs as they are ejected from the mouth. This variation results in a visibly different short- and long-term evolution of the puff, i.e., during, immediately following, and well after the puff is ejected. Variations in droplet distribution at the time of ejection could similarly have a profound impact on the droplet trajectories and long-time droplet behavior. In essence, even nearly similar puffs may have a substantially different evolution from one realization to another.

Three of the most important parameters of an ejected puff are its volume, momentum, and buoyancy, which in turn are also related to the mass, velocity, and temperature of the puff. In the case of a real cough or sneeze or their laboratory approximations, the puff is not instantaneously ejected. The ejection process extends over a finite time period, during which the ejection is characterized by the time history of volume flow rate. Another important parameter is the angle at which the puff is ejected. In most laboratory experiments and numerical simulations of coughing, sneezing, or talking, the puff is ejected from a stationary source and oriented along the horizontal direction, i.e., normal to the gravity field.^7,11,12^ In reality, however, the orientation of such events varies with time, which makes the puff and the droplet dynamics far more complex.

Aside from variations in the puff ejection process, ambient conditions also play an essential role. Such conditions include the temperature and humidity of both the ejected puff of air as well as the surrounding ambient air. The temperature difference between the puff and the ambient influences both the global motion of the puff through the buoyancy force, and the droplet trajectory, by increasing or decreasing the rate of droplet evaporation.^7,13,14^ Ambient humidity also has a strong influence on the droplet evaporation rate and its lifetime.^7,14^ Furthermore, a cross breeze or an elevated level of ambient turbulence^15^ can help to advect or diffuse a puff especially when the speed and turbulence level of the puff are comparable to or decay below those of the ambient. Apart from all the above factors, additional variability may be induced by the ambient room size,^16^ puff ejection frequency, i.e., consecutive coughs or sneezes,^17^ the use of face shields or masks, as well as other parameters.^18^

The puff dynamics from a cough or sneeze is of interest to the scientific community and the general public alike, especially during a health pandemic. To help reduce the rate of infection from airborne transmission, physical distancing guidelines suggest a separation distance of 6 ft between individuals. However, due to the huge variability in the nature of the puff and the ambient into which it is ejected, recent studies have shown that there are three different scenarios under which the ejected droplets and the viruses contained within can spread to distances much greater than 6 ft:^15,19,20^ (i) larger droplets ejected at very high speed in a violent cough or sneeze can ballistically travel and settle on surfaces that are at distances farther than 6 ft, (ii) in the case of an intense cough or sneeze, the ejected puff can propagate forward to distances larger than 6 ft, while carrying the small droplets that remain suspended within, and (iii) even in cases where the puff is not strong enough to travel farther from the source, the smaller ejected droplets that have evaporated to become droplet nuclei stay afloat for a long time to be carried and diffused over great distances by the ambient flow. In fact, recent laboratory experiments and direct measurements showed that the virus may spread to more than 7 m (approximately 21 ft) from the infected individual.^21,22^ Such measurements highlight the importance of airborne transmission, which represents a mechanism through which the virus can spread over distances much longer than a few meters. It also indicates that the 6 ft guideline, which was established in the 1930s,^23^ may not be adequate under certain conditions.

There is a growing number of experimental and numerical studies investigating the mechanisms of direct contact and airborne routes. Aside from the technical challenges usually encountered in laboratory experiments and high-quality measurements, the involvement of a human subject adds additional complications on safety and repeatability.^12,24^ Such complications are absent in numerical simulations, which have made them a popular tool for the study of puff and droplet dynamics. Many studies have been conducted using direct numerical simulations (DNS), large eddy simulations (LES), and Reynolds-averaged Navier–Stokes simulations (RANS).^7,15,19,25–27^ These simulations have proved useful in complementing laboratory experiments and visualizing the spreading of the micrometer-sized droplet nuclei over long distances and thereby highlighting the droplet transport process that is otherwise invisible to the naked eye. The simulations also allow exploration of different scenarios and investigation of the effects of varying the puff, droplet, and ambient parameters.

For example, Dbouk and Drikakis^19^ used RANS simulations to investigate the effect of wind speed on social distancing guidelines. They found that the virus-laden saliva droplets could travel up to 6 m with ambient wind speeds in the range of 4 to 15 km/h. On the other hand, they found that saliva droplets did not exceed a 2-m radius at zero wind speeds. Vuorinen *et al.*^27^ used LES to explore airborne transmission with regard to the number and distribution of infected individuals in public premises. They found that droplets in the range of 50 to 100 *μ*m may remain airborne for a few minutes, while smaller droplets (less than 20 *μ*m) may remain airborne for up to an hour. Chong *et al.*^7^ used DNS to illustrate the importance of considering the higher humidity of the puff exhaled together with the ejected liquid droplets and the role of ambient humidity as the outside air mixes with the puff fluid. They showed the importance of indoor ventilation and how humid ambient conditions can considerably extend virus-laden droplet lifetime.

The above simulations face important challenges and difficulties of a different nature than those encountered in laboratory experiments. DNS are the most accurate since they resolve all the fluid scales, but are prohibitively expensive since the Reynolds number of even a modest cough or sneeze is quite large requiring a very fine grid and a correspondingly small time step. LES simulations, where the large scale motion is resolved and sub-grid motion is modeled, are the next best option in terms of accuracy. Even though LES is more affordable than DNS, the simulations are still computationally demanding and require the use of sub-grid closure models.

While the aforementioned simulations have been quite useful in extending our understanding of the complex problem, an exhaustive coverage of all the possible scenarios of the puff and droplet generation is prohibitive due to the very large parameter space of the problem. A theoretical multiphase flow framework has recently been advanced^1^—it addressed the problem comprehensively starting from the formation of the droplet spectra and its ejection with the puff, continuing with the forward propagation of the puff along with the droplets which are undergoing simultaneous evaporation and gravitational settling, and ending with the inhalation of the droplet nuclei by the receiving host while accounting for the inhalation and filtration efficiency of protective devices such as masks. Starting from an initial droplet distribution ejected by the infected host, the theory predicted the concentration of droplet nuclei that remained within the puff, along with the location and size of the puff, as a function of time after the ejection event. The theory can thus be used to readily predict the probability of inhaling virus-laden droplet nuclei under a wide variety of ejection conditions, for a wide range of values of puff, droplet, and ambient parameters.

The purpose of the present simulations is to critically evaluate the theoretical framework presented in Balachandar *et al.*^1^ This will be accomplished with the following three steps:

• The simplified theoretical model was made possible by a number of assumptions: (i) the buoyancy effect on the puff is important only at late times when the puff velocity has decayed below the ambient velocity fluctuations; (ii) the puff can be approximated as a spherical volume whose size increases with time due to entrainment; (iii) the droplets are sufficiently small that their velocity can be approximated as the sum of the local fluid velocity and the still-fluid settling velocity,^28,29^ and the temperature may also be approximated using a similar equilibrium Eulerian assumption; and (iv) droplet evaporation follows an effective *d*^2^-law. The validity of these assumptions will be evaluated.
• The self-similar puff model involves a few closure parameters, whose values cannot be determined by the theory alone and thus must be empirically obtained from either experiments or simulations. These closure parameters include (i) the entrainment coefficient, (ii) the drag coefficient of the puff, (iii) the virtual origin location as measured from the source (i.e., distance from the mouth), and (iv) the virtual time of injection. We expect these closure parameters to depend on the integral puff parameters (i.e., puff volume and puff momentum). Values of these closure parameters, extracted from the simulations for a few different combinations of integral puff parameters, will be presented.
• The theoretical model yielded concrete results on (i) the power-law evolution of the puff size and puff location as a function of time, (ii) the largest droplet size that remains within the puff as a function of time, (iii) the largest, fully evaporated droplet size that remains within the puff, i.e., droplet nucleus, and (iv) the droplet size spectrum within the puff at later times. The accuracy of these predictions will be evaluated with the simulation results.

The rest of the paper is organized as follows. In Sec. II, we discuss the governing equations for the fluid and droplet phases in the Euler–Lagrange (EL) framework. In Sec. III, we lay out the simulation details for the considered cases. The results for the puff and droplet dynamics are presented and discussed in Secs. IV and V, respectively. Conclusions are drawn in Sec. VI.

## II. GOVERNING EQUATIONS OF EULER-LAGRANGE LES

In this section, we present the mathematical model of the framework and the numerical methodology used in the simulations. Lowercase and uppercase variables denote Eulerian grid-based and Lagrangian particle-based quantities, respectively. For example, the fluid velocity in the Eulerian frame of reference is denoted by the field u(x,t), while particle velocity in the Lagrangian frame of reference is denoted by V(t).

**FIG. 1.**
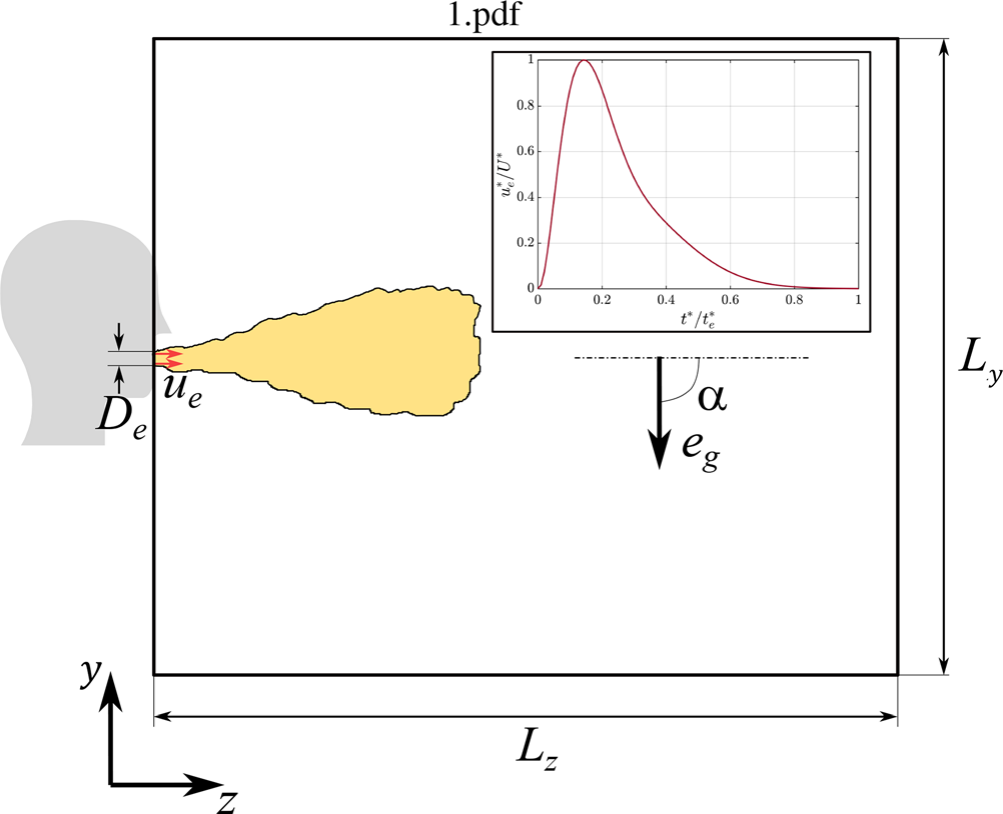
Schematic of the computational domain.

Figure 1 shows a schematic representation of the numerical setup. The ejected puff of fluid along with the droplets is allowed to enter the cuboidal computational domain through a circular opening of diameter $L_{e*}$. The ejection process is taken to be time dependent and lasts for a duration of $t_{e*}$. Based on the experimental observations,^30^ the ejection velocity of the puff increases rapidly to reach a peak velocity of $U_{*}$ and then slowly decays to zero after a time period of $t_{e*}$. Thus, the ejection process evolves as defined by the time-dependent ejection velocity $u_{e*}(t_{*})$ (24). The ejected puff is at a temperature $T_{e*}$, which is typically higher than the ambient temperature $T_{a*}$, both of which will be taken to be constant. The three *integral parameters* that characterize the ejected puff are its volume, momentum, and buoyancy, which are given by

$$Q_{e*}=\frac{\pi L_{e*}^{2}}{4}\int_{0}^{t_{e*}}u_{e*}(\tau_{*})\;d\tau_{*},$$ (1)

$$M_{e*}=\frac{\pi L_{e*}^{2}}{4}\rho_{a*}\int_{0}^{t_{e*}}u_{e*}^{2}(\tau_{*})\;d\tau_{*},$$ (2)

$$B_{e*}=\frac{\pi L_{e*}^{2}}{4}(\rho_{a*}-\rho_{e*})\;g_{*}\int_{0}^{t_{e*}}u_{e*}(\tau_{*})\;d\tau_{*},$$ (3)

where $\rho_{e*}$ is the density of the ejected fluid and $\rho_{a*}$ is the density of the ambient air. The values of these and other simulation parameters can be found in Table I. The temperature difference between the ejected puff and the ambient (i.e., the difference $T_{e*}-T_{a*}$) is typically of the order of ten degrees centigrade. The corresponding density difference of air is quite small and therefore we will make the Boussinesq approximation that $\rho_{e*}\approx \rho_{a*}$ everywhere except in the buoyancy term. The Boussinesq approximation is the reason the momentum $M_{e*}$ definition uses the ambient density, while the buoyancy definition retains the density difference. Here and throughout the manuscript, an asterisk denotes a dimensional quantity, and all other quantities are to be understood as nondimensional.

**TABLE I.** List of parameters held fixed across all simulations. When appropriate, parameters are given in their dimensional (denoted by an asterisk) and nondimensional forms.

Mouth diameter	$L_{e*}=2.26$ cm	Mouth area	$A_{e*}=4.00$ cm^2^
Ambient temperature	$T_{a*}=20\;{}^{\circ}$ C	Ejection temperature	$T_{e*}=35\;{}^{\circ}$ C
Ambient density	$\rho_{a*}=1.204$ kg/m^3^	Ejection density	$\rho_{e*}=1.146$ kg/m^3^
Ambient kinematic viscosity	$\nu_{a*}=1.516\times 10^{-5}$ m^2^/s	Water density	$\rho_{w*}=996.12$ kg/m^3^
Ambient thermal diffusivity	$\alpha_{a*}=2.17\times 10^{-5}$ m^2^/s	Ambient thermal conductivity	$k_{a*}=2.59\times 10^{-2}$ W/(m K)
Ambient specific heat	$C_{pa*}=1.013\times 10^{3}$ J/(kg K)	Water latent heat of vaporization	$L_{*}=2.453\times 10^{6}$ J/kg
Specific heat of water	$C_{pw*}=4.182\times 10^{3}$ J/(kg K)	Gravitational acceleration	$g_{*}=9.81$ m/s^2^
Number of ejected droplets	*N_e_* = 61650	Volume of ejected droplets	$v_{e*}=1.316\times 10^{-5}$ L $v_{e}=1.14\times 10^{-3}$
Smallest ejected droplet diameter	$D_{1*}=1\;\mu$m $D_{1}=4.42\times 10^{-5}$	Largest ejected droplet diameter	$D_{2*}=1$ mm $D_{2}=4.42\times 10^{-2}$
Droplet distribution coefficient	$B_{p*}=6.1$ cm $B_{p}=2.74$		

The governing equations of the fluid and the droplet phases will be presented in dimensionless form using the mouth diameter $L_{e*}$ as the length scale, the peak ejection velocity $U_{*}$ as the velocity scale, and $L_{e*}/U_{*}$ as the time scale. The nondimensional temperature perturbation is defined as $T=(T_{*}-T_{a*})/(T_{e*}-T_{a*})$. As the puff fluid entrains and mixes with ambient fluid, the temperature of the fluid within the computational volume will range from $T_{a*}$ to $T_{e*}$, and accordingly the nondimensional temperature will range from zero in the ambient to unity in the unmixed puff fluid.

It was shown in Balachandar *et al.*^1^ that temperature differences of O(10 °C) between the ejected puff and the ambient do not significantly alter the dynamics of the puff. The buoyancy effect of the temperature difference will begin to play a role only at later times when the puff velocity has sufficiently fallen to small values. For example, the puff behaves as a momentum-dominated jet-like flow, and only at times larger than a transition time $t_{tr*}$ when the puff velocity is sufficiently reduced, do ambient turbulence and buoyancy effects begin to play a dominant role in further development. This transition time can vary from a few seconds to a few tens of seconds depending on the initial momentum of the puff.^1^ Nevertheless, the present simulations will include the energy equation of the fluid and thereby account for the late-time buoyancy effects.

The entire spectrum of droplets that is ejected with the puff is assumed to follow the Pareto size distribution^1^ with the largest and the smallest ejected droplets to be of size $D_{1*}$ and $D_{2*}$, respectively. The Pareto distribution of the ejected droplets is given by the power-law $N_{e}(D_{*})=B_{p*}/D_{*}^{2}$, where $N_{e}(D_{*})dD_{*}$ denotes the number of droplets ejected over the size range $\left(D_{*}-dD_{*}/2\right)$ and $\left(D_{*}+dD_{*}/2\right)$, and $B_{p*}$ is the Pareto pre-factor. Thus, the total number and volume of ejected droplets are given by

$$N_{e}=\int_{D_{1*}}^{D_{2*}}N_{e}\;dD_{*}=\frac{B_{p*}(D_{2*}-D_{1*})}{D_{1*}D_{2*}},$$ (4)

$$v_{*e}=\int_{D_{1*}}^{D_{2*}}N_{e}\left(\frac{\pi }{6}D_{*}^{3}\right)\;dD_{*}=\frac{\pi B_{p*}(D_{2*}^{2}-D_{1*}^{2})}{12}.$$ (5)

We will assume the droplets to be uniformly, but randomly, distributed within the ejected puff such that the corresponding average number density of droplets (i.e., number of droplets per unit volume) and average droplet volume fraction are obtained as

$$\chi_{e*}=\frac{N_{e}}{Q_{e*}}\;\text{and}\;\phi_{e*}=\frac{v_{e*}}{Q_{e*}}.$$ (6)

Here, $N_{e}$ and $v_{e*}$ are the total number and volume of ejected droplets. The droplets are also ejected in a time-dependent manner over the time interval from 0 to $t_{e*}$ while maintaining the number density and volume fraction of ejected droplets at $\chi_{e*}$ and $\phi_{e*}$, respectively.

### A. Fluid phase

The Reynolds number of the flow can be defined in different ways, and two different definitions are provided below:

$${\text{Re}}_{\mathrm{inj}}=\frac{L_{e*}\;U_{*}}{\nu_{a*}},$$ (7)

$${\text{Re}}_{pf}=\frac{Q_{e*}^{1/3}\;(M_{e*}/(\rho_{a*}Q_{e*}))}{\nu_{a*}},$$ (8)

where the injection Reynolds number ${\text{Re}}_{\mathrm{inj}}$, which is based on mouth opening and maximum injection velocity better characterizes the flow during the ejection process, while the Reynolds number ${\text{Re}}_{pf}$ is better suited to characterize the puff as a whole as it propagates forward into the ambient. Irrespective of the definition, the Reynolds number of even modest ejections is sufficiently large and the associated Kolmogorov length scale is *O*(100) *μ*m. The resolution of the entire range of turbulent length (and time) scales poses a great challenge, even without accounting for the smaller-sized droplets. The very wide range of length scales, from the sub-micron droplets to distances over a meter that the puff travels, prevents droplet-resolved simulations of a cough or a sneeze. Therefore, in the present work we pursue an EL-LES approach of the puff with the droplets taken into account through the point-particle model.

The typical volume fraction of droplets within the puff at ejection is lower than $10^{-4}$. The droplet volume fraction within the puff will further decrease over time due to (i) the rapid evaporation of droplets, (ii) the fallout of larger droplets, and (iii) the enlargement of the puff by ambient fluid entrainment. Even with the large water-to-air density ratio, the mass loading of droplets within the puff is quite small at ejection and continues to decrease over time. Shortly after ejection, the velocity of the droplets can differ from that of the fluid, and this difference can be as large as O(10) m/s for large droplets, since they are ballistic. However, such droplets are few in number, and they quickly overshoot and exit out of the puff. The droplets that stay within the puff rapidly evaporate, and they quickly equilibrate to the puff velocity. In essence, to the leading order, the momentum coupling of the droplets back on the fluid is quite small.

The temperature of the ejected droplets can differ from that of the ejected fluid, and both these temperatures will differ from that of the ambient. As the droplets rapidly evaporate, the thermal energy needed for the phase change comes through heat transfer from the surrounding fluid. The analysis of Balachandar *et al.*^1^ shows that the resulting droplet temperature is only a few degrees lower than the surrounding fluid. The aforementioned heat transfer to the evaporating droplets, however, lowers the temperature in the surrounding fluid and brings it closer to the cooler ambient temperature. The decreased temperature of the puff and the increased water vapor content within the puff alter the buoyancy effect, which as discussed above, remains small especially shortly after ejection. The gas-phase governing equations are the incompressible Navier–Stokes equations with the Boussinesq approximation of accounting for the density difference only in the buoyancy term. In LES, the gas-phase velocity **u** is spatially smoothened with a filter function $G(x,x^{\prime })$ as

$$u_{f}(x,t)=\int G(x,x^{\prime })\;u(x\prime ,t)dx\prime ,$$ (9)

where the integral is over a large volume centered around the point **x**. The filter function rapidly decreases for increasing |x−x′| and has the property $\int G(x,x^{\prime })\;dx\prime =1$. It is designed to filter out all fluid velocity variations smaller than a chosen length scale, which will be chosen to be slightly larger than the grid spacing. Thus, all the filtered length scales are numerically resolved in the LES simulation. In the context of droplet-laden flow, the above filtering process has the advantage that the filtered velocity $u_{f}$ is defined over the entire volume including the region occupied by the droplets. On the other hand, the unfiltered gas-phase velocity **u** is defined only in the region outside the droplets. The filtered pressure and thermal fields of the gas-phase can be similarly defined. The filtered gas-phase governing equations have been rigorously derived in the context of EL multiphase flow simulations.^31,32^ In the present limit of very low droplet volume fraction, the governing equations can be further simplified to obtain the following nondimensional equations:

$$\nabla \cdot u_{f}=0,$$ (10)

$$\frac{\partial u_{f}}{\partial t}+u_{f}\cdot \nabla u_{f}=-\nabla p_{f}+\left(\frac{1}{{\text{Re}}_{\mathrm{inj}}}+\nu_{t}\right)\nabla^{2}u_{f}+T_{f}g\prime e_{g}-\sum_{l}F_{l}\prime \;G(x,X_{l}),$$ (11)

$$\frac{\partial T_{f}}{\partial t}+u_{f}\cdot \nabla T_{f}=\left(\frac{1}{Pe}+\frac{\nu_{t}}{\text{Pr}}\right)\nabla^{2}T_{f}-\sum_{l}q_{l}\prime \;G(x,X_{l}),$$ (12)

where subscript *f* indicates filtered variables and *p_f_* is the pressure after subtraction of the hydrostatic component. Furthermore, the reduced gravity is defined as

$$g\prime =\frac{\rho_{e*}-\rho_{f*}}{\rho_{f*}}\frac{g_{*}D_{e*}}{U_{*}^{2}},$$ (13)

and $e_{g}$ is a unit vector pointing in the direction of gravity. We choose the *z* axis of the computational domain to be along the direction of ejection, and the *x* axis to be along the transverse horizontal direction (normal to gravity). Except in cases where the puff is ejected vertically downward or upward, the gravity vector will have a component along the *y* axis. In the present study, the *y* axis is aligned with the gravity vector since the puff is ejected horizontally (i.e., α=π/2 as seen in Fig. 1). In general, the puff direction is given by the angle *α*, which is measured from the gravity vector. The unit vector $e_{g}$ is thus $e_{g}=(0,-\text{sin}\;\alpha ,\;\text{cos}\;\alpha )$.

The filtering process introduces an unknown sub-grid Reynolds stress term into the filtered momentum equation, which has been closed with the eddy viscosity model, where the nondimensional turbulent eddy viscosity *ν_t_* is obtained using the dynamic Smagorinsky model.^33–35^ In the energy equation, the filtering operation similarly introduces the sub-grid heat flux term which has been closed with the gradient diffusion model, where the nondimensional diffusion coefficient is taken to be $\nu_{t}/\text{Pr}$ with the Prandlt number of air fixed at Pr=0.7. In general, in multiphase LES, the sub-grid stress and heat flux will receive contributions both from the turbulence cascade feeding energy to the filtered small scales, as well as from pseudo turbulence generated by the wakes of the droplets. In the present problem, due to the very low volume fraction of the suspended droplets, the latter contribution is negligible and the classical single-phase LES closure is adequate.

In the last term on the right-hand side of the momentum and energy equations, $F_{l}^{\prime }$ is the nondimensional hydrodynamic perturbation force on the *l*th droplet and $q_{l}^{\prime }$ is the scaled perturbation heat transfer to the *l*th droplet, whose center is at $X_{l}$. The force and heat transfer of each droplet are fed back to the fluid (with a negative sign), and the filter function converts these Lagrangian quantities into Eulerian fields. However, as noted previously, these feedback effects of the droplets on the gas-phase are quite weak and have been ignored in the present simulations. We have confirmed that their neglect, from both the momentum and thermal equations, do not alter the results to be discussed below.

### B. Particle phase

Each ejected droplet is individually tracked. For the dispersed phase, droplet dynamics are dependent on different force components. For a dilute two-phase flow, direct (collision) and indirect (fluid-mediated) interactions within the dispersed phase are negligible. The governing equations of the mass, position, velocity, and temperature of the *l*th droplet in nondimensional form are

$$\frac{d}{dt}\left[\begin{array}{c} m_{l}\\ X_{l}\\ V_{l}\\ T_{l} \end{array}\right]\;=\left[\begin{array}{c} -\pi D_{l}\;\text{Nu}\;\;\text{ln}\;(1+B_{m,l})\;\frac{1-\psi_{l}}{{\text{Re}}_{\mathrm{inj}}\;\text{Sc}}\\ V_{l}\\ F_{l}/m_{l}\\ q_{l}/(m_{l}\;C_{r})+(L/m_{l})\;dm_{l}/dt \end{array}\right].$$ (14)

In the mass conservation equation, first equation in (14), $m_{l}=\pi D_{l}^{3}\rho /6$ is the mass of the *l*th droplet and $\rho =\rho_{w*}/\rho_{a*}$ is the density ratio of water ( $\rho_{w*}$) to ambient air ( $\rho_{a*}$). The right-hand side corresponds to the nondimensional evaporation rate of the *l*th droplet in question. Here, $\text{Nu}=2+0.6{\text{Re}}_{p}^{1/2}{\text{Pr}}^{1/3}$ is the Nusselt number and $B_{m,l}=(Y_{l}-Y_{f@l})/(1-Y_{l})$ is the Spalding mass number, where *Y_l_* is the mass fraction of water vapor at the surface of the *l*th droplet and $Y_{f@l}$ is the mass fraction of water vapor in the surrounding fluid at the droplet location. The Schmidt number is defined as $\text{Sc}=\nu_{a*}/D_{a*}$, where $D_{a*}$ is the diffusion coefficient of water vapor in air.

A droplet ejected during a cough or sneeze is not made up of pure water. It contains salts, mucus material, viruses, and other nonvolatile particulate matters. The presence of nonvolatile matter is known to reduce the evaporation rate of water and this effect is empirically modeled as a correction factor, where $\psi_{l}=\psi_{0}D_{l0}^{3}/D_{l}^{3}$ is the instantaneous volume fraction of the nonvolatiles in the *l*th droplet. In this expression, *ψ*_0_ is the initial fraction of nonvolatiles in the droplet at the time of ejection before evaporation and $D_{l0}$ is the diameter of the *l*th droplet at the time of ejection. As evaporation proceeds, the volume fraction of nonvolatiles *ψ_l_* quickly increases from its initial value of *ψ*_0_, and once it reaches its upper limit of unity, all liquid would have evaporated, and the droplet becomes a droplet nucleus. It must be pointed out that even a small amount (by volume) of nonvolatile material in the ejected droplet, say $\psi_{0}=1\%$, will result in a final droplet nucleus of diameter about 21% of the initial diameter. The equation of mass evolution can be rewritten by defining an effective evaporation coefficient $k\prime_{st}=4D_{a*}{\text{Nu}}_{st}\;\text{ln}\;(1+B_{m,l})/\rho$, where ${\text{Nu}}_{st}=2$ is the steady state Nusselt number. With this, the mass balance can be expressed in terms of the time evolution of droplet diameter as

$$\frac{dD_{l}}{dt}=-k\prime_{st}\frac{\text{Nu}}{2\;{\text{Nu}}_{st}}\frac{1}{D_{l}}\left(1-\psi_{0}\frac{D_{l0}^{3}}{D_{l}^{3}}\right).$$ (15)

Though the Spalding mass number *B_m_* is a function of droplet temperature and the local mass fraction of water in the surrounding fluid, we will assume this variation to be weak and take $k\prime_{st}$ to be constant.

In the momentum equation, the total force $F_{l}$ acting on the *l*th droplet is the summation of the undisturbed ( $F_{\text{un},l}$), quasi-steady ( $F_{\text{qs},l}$), added-mass ( $F_{\text{am},l}$), and gravity-buoyancy ( $F_{g,l}$) forces, i.e.,

$$F_{l}=F_{\text{un},l}+\underset{F_{l}^{\prime }}{\underset{︸}{F_{\text{qs},l}+F_{\text{am},l}}}+F_{g,l}.$$ (16)

The undisturbed flow force is exerted even in the absence of the droplet, and thus, only the sum of the quasi-steady and the added-mass forces represents the hydrodynamic perturbation force due to the presence of the droplet. We note that their sum, denoted as $F_{l}^{\prime }$, and not the individual contribution of each term, is fed back to the gas-phase. In the above, we have ignored the viscous history force. In fact, due to the large value of density ratio *ρ*, only the quasi-steady and gravitational forces are of importance in the present problem. The closure expressions of the above nondimensional forces are

$$F_{\text{qs},l}=\frac{3\pi D_{l}}{\text{Re}}\left(u_{f}\left(X_{l}\right)-V_{l}\right)\Phi \left({\text{Re}}_{l}\right),$$ (17)

$$F_{\text{un},l}=V_{l}{\frac{Du_{f}}{Dt}|}_{x=X_{l}},$$ (18)

$$F_{\text{am},l}=\frac{1}{2}V_{l}\left({\frac{Du_{f}}{Dt}|}_{x=X_{l}}-\frac{dV_{l}}{dt}\right),$$ (19)

$$F_{g,l}=V_{l}\;g\prime \;e_{g},$$ (20)

where $V_{l}=\pi D_{l}^{3}/6$ is the volume of the *l*th droplet. The Reynolds number of the *l*th droplet is given in terms of the injection Reynolds number and the droplet relative velocity as ${\text{Re}}_{l}={\text{Re}}_{\mathrm{inj}}|u_{f}\left(X_{l}\right)-V_{l}|D_{l}$. $\Phi ({\text{Re}}_{l})=1+0.15{\text{Re}}_{l}^{0.687}$ is the finite Reynolds number drag correction, which depends on the droplet Reynolds number. In the above force expressions, the filtered LES fluid velocity $u_{f}$ and the total fluid acceleration $Du_{f}/Dt$ are evaluated at the location of the *l*th droplet through interpolation.

In the energy equation (12), the first term on the right-hand side is the heat transfer to the droplet from the surrounding fluid and the second term is associated with the latent heat of vaporization. The heat transfer to the *l*th droplet is given by^36^

$$q_{l}=\frac{\pi k_{a*}D_{l*}\text{Nu}}{\rho_{a*}C_{pa*}U_{*}L_{e*}^{2}}\frac{\;\text{ln}\;(1+B_{m,l})}{B_{m,l}}(T_{f@l}-T_{l}),$$ (21)

where $k_{a*}$ is the thermal conductivity of air, $C_{pa*}$ is the specific heat of air, and $C_{r}=C_{pw*}/C_{pa*}$ is the ratio of specific heat of water to that of air. Also, $L=L_{*}/(C_{pw*}(T_{e*}-T_{a*}))$, where $L_{*}$ is the latent heat of evaporation of water vapor. In the present study, due to only a small variation in the droplet and air temperatures, we take all thermodynamic and transport properties of the droplet and air to be constant. Since we ignore in (14) the thermal back coupling to the fluid from the droplets, and we assume a constant $k\prime_{st}$, the droplet temperature equation decouples from the rest of the governing equations. Thus, the precise value of parameters such as *L* and *C_r_* are only important for droplet temperature and are unimportant for the puff dynamics and droplet evolution. As shown in Balachandar *et al.*,^1^ the temperature equation in (14) can be analytically solved using the equilibrium Eulerian approach^28,29^ in the limit of small droplet thermal timescale to obtain an explicit leading order equation for the droplet temperature in terms of the local fluid temperature.

## III. NUMERICAL METHODOLOGY AND SIMULATION DETAILS

The gas-phase LES equations are solved using a highly scalable spectral element solver^37,38^ in a domain of size $L_{x}\times L_{y}\times L_{z}$ along the transverse, vertical, and flow directions, respectively (see Fig. 1). The particular values chosen for *L_x_*, *L_y_*, and *L_z_* depend on the intensity of ejection measured in terms of ${\text{Re}}_{\mathrm{inj}}$ and are listed in Table II along with other simulation parameters for the different cases considered. The domain is discretized using $N_{x}\times N_{y}\times N_{z}$ hexahedral elements with *N*^3^ Gauss–Lobatto–Legendre (GLL) grid points within each element. A Dirichlet boundary condition for temperature and velocity is imposed at the inlet plane *z *=* *0, while open boundary conditions are applied at the other five boundaries.^39^

**TABLE II.** Details of the numerical simulations. $U_{*}$ is the peak ejection velocity, $t_{e*}$ is the ejection duration, $Q_{e*}$ is the puff volume (1), $M_{e*}$ is the puff momentum (2), $B_{e*}$ is the puff buoyancy (3), *Re_inj_* is the injection Reynolds number (7), *Re_pf_* is the puff Reynolds number (8), $k\prime_{st}$ is the droplet evaporation coefficient. Fewer grid points are needed along the *x* and *y* directions since these points are clustered near the center of the domain.

Simulation	$U_{*}$ (m/s)	$t_{e*}$ (s)	$Q_{e*}$ (m^3^)	$M_{e*}$ (kg m/s)	$B_{e*}$ (N)	${\text{Re}}_{\mathrm{inj}}$	${\text{Re}}_{pf}$	Domain size $L_{x*},L_{y*},L_{z*}$ (m, m, m)	Grid resolution $N_{x},N_{y},N_{z}$	$k\prime_{st}$
Q10V20a	30.7	0.29	$10^{-3}$	2.41 $\times 10^{-2}$	17.5	4.57 $\times 10^{4}$	1.32 $\times 10^{5}$	1.81, 1.81, 1.81	120, 120, 960	3.6 $\times 10^{-7}$
Q10V20b	30.7	0.29	$10^{-3}$	2.41 $\times 10^{-2}$	17.5	4.57 $\times 10^{4}$	1.32 $\times 10^{5}$	1.81, 1.81, 1.81	120, 120, 960	3.6 $\times 10^{-7}$
Q10V20c	30.7	0.29	$10^{-3}$	2.41 $\times 10^{-2}$	17.5	4.57 $\times 10^{4}$	1.32 $\times 10^{5}$	1.81, 1.81, 1.81	120, 120, 960	3.6 $\times 10^{-7}$
Q10V20d	30.7	0.29	$10^{-3}$	2.41 $\times 10^{-2}$	17.5	4.57 $\times 10^{4}$	1.32 $\times 10^{5}$	1.81, 1.81, 1.81	120, 120, 960	3.6 $\times 10^{-7}$
Q10V20e	30.7	0.29	$10^{-3}$	2.41 $\times 10^{-2}$	17.5	4.57 $\times 10^{4}$	1.32 $\times 10^{5}$	1.81, 1.81, 1.81	120, 120, 960	1.4 $\times 10^{-9}$
Q10V20f	30.7	0.29	$10^{-3}$	2.41 $\times 10^{-2}$	17.5	4.57 $\times 10^{4}$	1.32 $\times 10^{5}$	1.81, 1.81, 1.81	120, 120, 960	1.4 $\times 10^{-11}$

The Lagrangian droplets are solved using the highly scalable point-particle library ppiclF.^40–42^ The interpolation of the Eulerian fluid quantities to the Lagrangian droplet location is achieved using the highly efficient Barycentric interpolation technique, which preserves spectral accuracy.^41^ The droplet injection is only through the circular inlet at the *z *=* *0 plane, which will be described below. At all other surfaces, a droplet can only leave the computational domain, in which case it is removed from further consideration. Only a few very large droplets that are injected at the highest velocities and do not evaporate fast enough end up exiting the computational domain. As will be demonstrated in the results, most of the droplets either remain within the puff or within the computational domain.

Droplet injection is achieved in four steps: (i) the number of droplets to be injected over a short time span *δt* is specified, (ii) each of the injected droplets is then randomly placed within a small cylindrical volume behind the injection plane, (iii) the diameter of the injected droplets is determined, and finally (iv) the initial injection velocity of the droplets is specified. These four injection steps will be briefly detailed below. The present work does not account for velocity fluctuations and correlations that may exist between the droplets and the fluid at the time of injection. A more comprehensive injection framework has been presented in Ref. 43 in the context of a mid-field spray simulation, where detailed gas-phase and droplet phase velocity measurements were available. With the availability of such detailed measurements in the context of coughs and sneezes, an improved injection model can be pursued.

The volume of injected fluid over the time span *δt* is $\pi \bar{u_{e}}\delta tL_{e*}^{3}/4$, where $\bar{u_{e}}$ is the average nondimensional injection velocity during this period. This yields the number of droplets to be injected during this time span to be

$$\chi_{e*}\frac{\pi }{4}\;\bar{u_{e}}\;\delta t\;L_{e*}^{3}.$$ (22)

The droplets injected during this time span are randomly placed within a cylindrical volume of unit diameter and length $\bar{u_{e}}\;\delta t$ adjacent to the inlet circular port. The diameter of injected droplets is then determined from the cumulative Pareto distribution as

$$D_{l0}=\frac{D_{1}}{1-(1-D_{1}/D_{2})R},$$ (23)

where $D_{1}=D_{1*}/L_{e*}$ and $D_{2}=D_{2*}/L_{e*}$ are the nondimensional minimum and maximum droplet diameters at injection and R∈(0,1) is a random number. The initial velocity of droplets is assumed to have the same magnitude as the instantaneous gas ejection velocity, namely $\left|V_{j}(t=0)|=|u_{e}\right|$, but with a normally distributed ejection angle, *θ_v_*, measured with respect to the ejection direction. We also assume zero initial circumferential velocity. To avoid unrealistic extreme values, we ignore the rare droplets whose initial angle of ejection is too large (i.e., $|\theta_{v}|>45{}^{\circ}$), which only account for less than 0.3% of the total number of ejected droplets.

The droplet laden puff that is ejected during a cough or a sneeze varies from one person to another and for the same individual from one cough or sneeze to another. As far as the puff is concerned, the variation includes a number of parameters such as puff volume, duration of ejection, mouth size, ejection velocity profile, ejection temperature, ejection angle, ambient temperature, and so on. Here we consider six simulations whose details are listed in Table II. As far as the puff dynamics is concerned, the six cases only vary by the small velocity and temperature perturbations at ejection. They are statistically identical to one another and constitute different realizations for the same case. However, two of the cases Q10V20e and Q10V20f have different droplet evaporation coefficients than the other four cases.

Another quantity of importance is the ejection velocity profile of the puff as it exits the mouth. This time-dependent profile is obtained from the experiments of Gupta *et al.*,^30^ who provided the following fit:

$$\begin{array}{c} u_{e*}\left(\frac{t_{*}}{t_{e*}}\right)=U_{*}\{a_{1}{\left(\frac{t_{*}}{t_{e*}}\right)}^{b_{1}}\text{ex}p\left[-c_{1}\left(\frac{t_{*}}{t_{e*}}\right)\right]\\ +H\left(\frac{t_{*}}{t_{e*}}-d_{1}\right)a_{2}{\left(\frac{t_{*}}{t_{e*}}-d_{1}\right)}^{b_{2}}e\text{xp}\left[-c_{2}\left(\frac{t_{*}}{t_{e*}}-d_{1}\right)\right]\}, \end{array}$$ (24)

where H is the Heaviside step function, and the values of the seven fitting coefficients are $a_{1}=962.42,\;b_{1}=2.34,\;c_{1}=16.24,\;d_{1}=0.173,\;a_{2}=32466,\;b_{2}=5.31$, and $c_{2}=20.5$. A plot of this ejection velocity is shown in Fig. 1. In all cases, random axial and radial velocity perturbations of 5% amplitude, compared to the peak injection velocity, were introduced to achieve faster turbulence transition and investigate variations across the different realizations. Similarly, random perturbations of 5% amplitude were added to the inlet temperature profile; however, these perturbations are of far lower significance compared to those introduced to the velocity profile.

## IV. EVOLUTION OF THE PUFF

### A. Structure of the puff

**FIG. 2.**
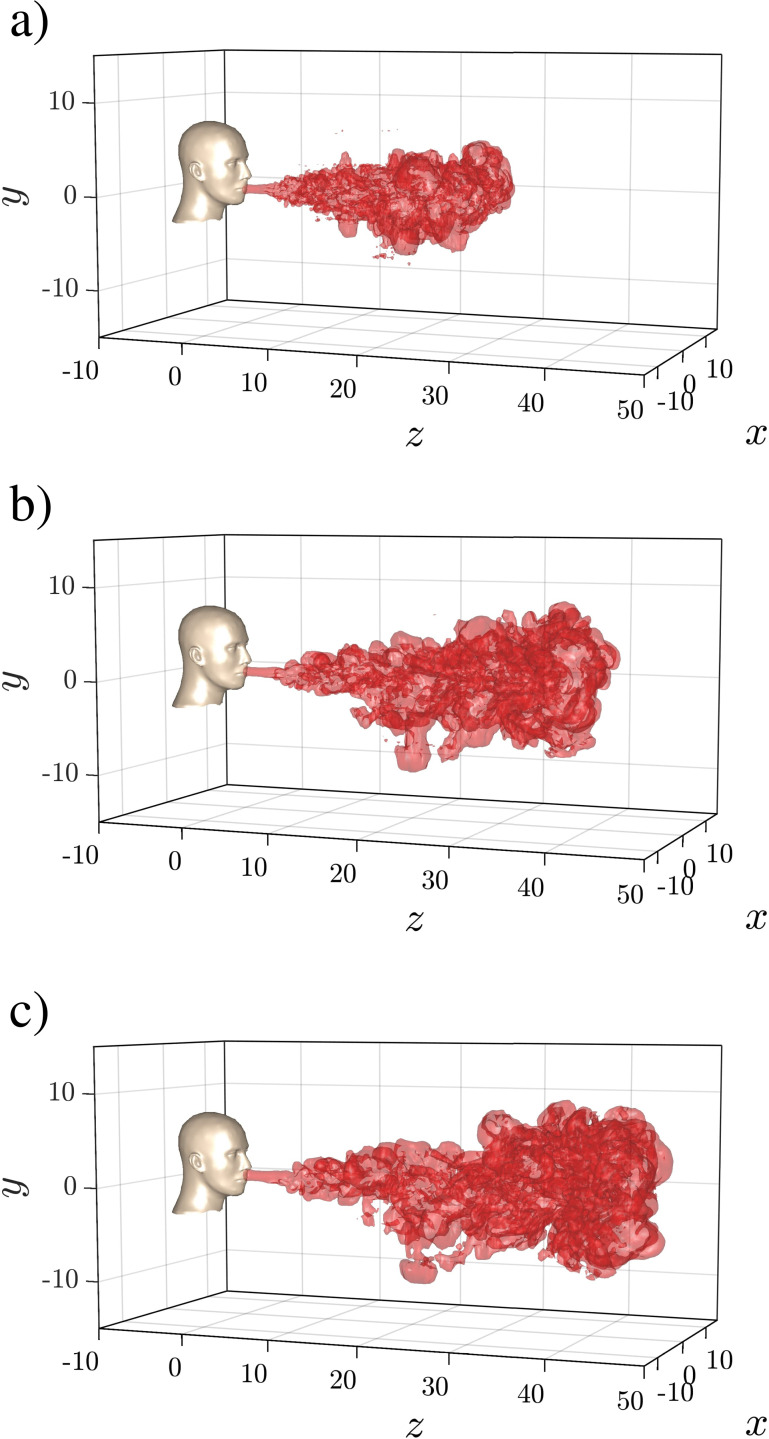
Three-dimensional structure of the turbulent puff for Case Q10V20d visualized using iso-surfaces of temperature $\left(T_{f}=0.01\right)$ (a) toward the end of the injection phase (*t *=* *243), (b) at an intermediate stage (*t *=* *728), and (c) at the end of the simulation (*t *=* *1213). The nondimensional length and time units correspond to approximately 2.26 cm and 0.77 ms.

In Fig. 2, we consider the 3D structure of the puff at three time instances, namely toward the end of injection (panel a), at an intermediate time after injection is complete (panel b), and near the end of the simulation (panel c). The structure of the puff is extracted using a temperature iso-surface of $T_{f}=0.01$. The structure of the puff is clearly indicative of the turbulent flow inside, except very close to the mouth area. As seen in the inset of Fig. 1, the ejection velocity is very small near the end of the ejection phase, which results in a quiescent tail at the end of the puff. This tail remains nearly stagnant and intact due to lack of mixing, unlike the rest of the puff which vigorously mixes with the ambient through entrainment. As we will see later, the number of ejected droplets during this late phase is quite small and therefore the tail is not of significance.

**FIG. 3.**
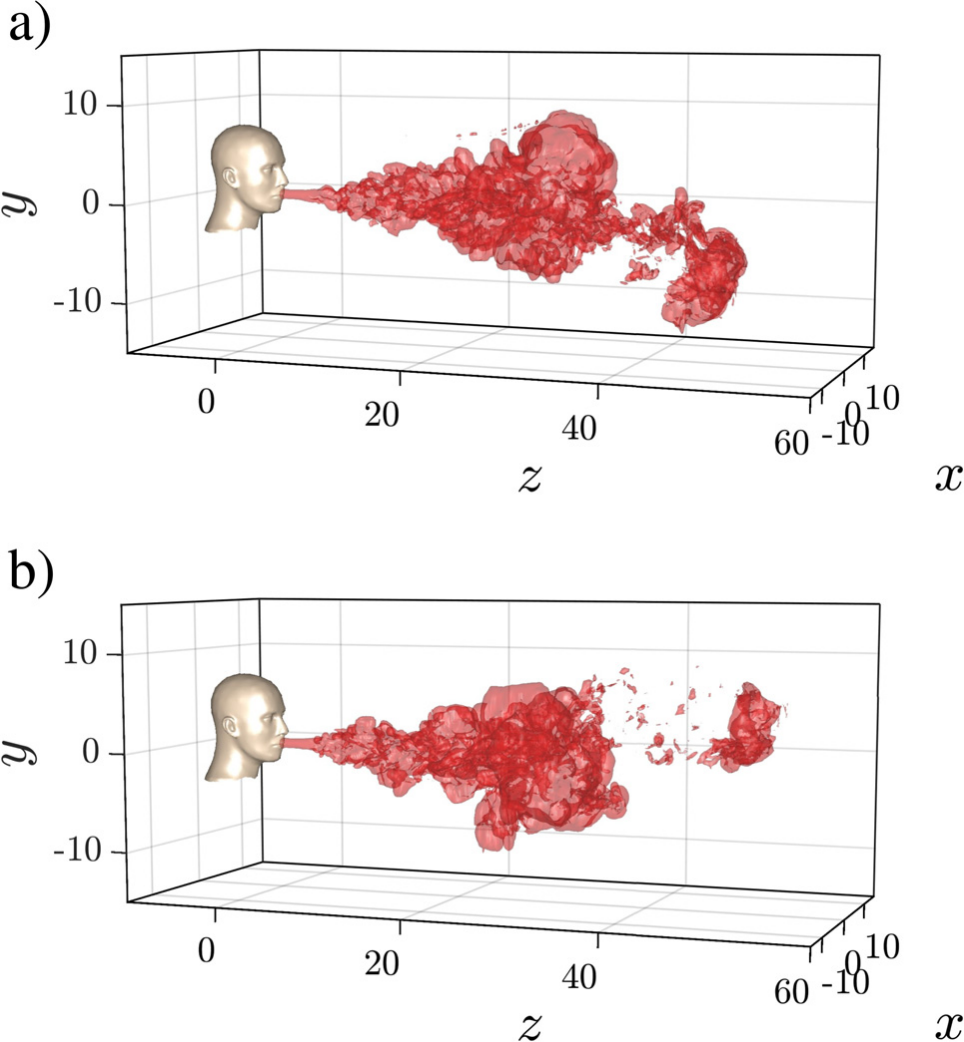
Three-dimensional structure of the turbulent puff for cases Q10V20b and Q10V20c visualized using iso-surfaces of temperature equivalent to 1% of the puff ejection temperature $\left(T_{f}=0.01\right)$ at *t *=* *728. Compare with the structure from Fig. 2 at the same time instance. The figure highlights the realization to realization variability. The nondimensional length and time units correspond to approximately 2.26 cm and 0.77 ms.

It is interesting to note that the small perturbations result in visibly different puff structures for the different realizations. More specifically as seen in Fig. 2 for Case Q10V20d, the puff remains fairly coherent as a single connected unit for the entire duration of the simulation, with only small fragments peeling off the main body of the puff. On the other hand, we observe from Fig. 3, for Case Q10V20b (respectively, Q10V20c) that while the bulk of the puff remains as a single connected unit, a small portion at the downstream end of the puff detaches from the main body and advances downward and to the right (respectively, upward and to the left). These detaching portions have a distinct vortex ring-like character that allows them to advance a little faster than the main body. We should note here that due to the axisymmetric nature of the puff there is equal probability for the detached portion to split off toward the right or the left of the domain. Furthermore, due to the weak influence of buoyancy in the early stages of the puff evolution, it is also likely that the detached portion would have an equal probability of splitting off toward the top or the bottom of the domain. That being said, we observe the main component of motion for the detached puff to remain along the puff direction (*z* axis). Except for Case Q10V20d, all puffs exhibited the detached vortex that advanced in a different direction from one realization to the other.

**FIG. 4.**
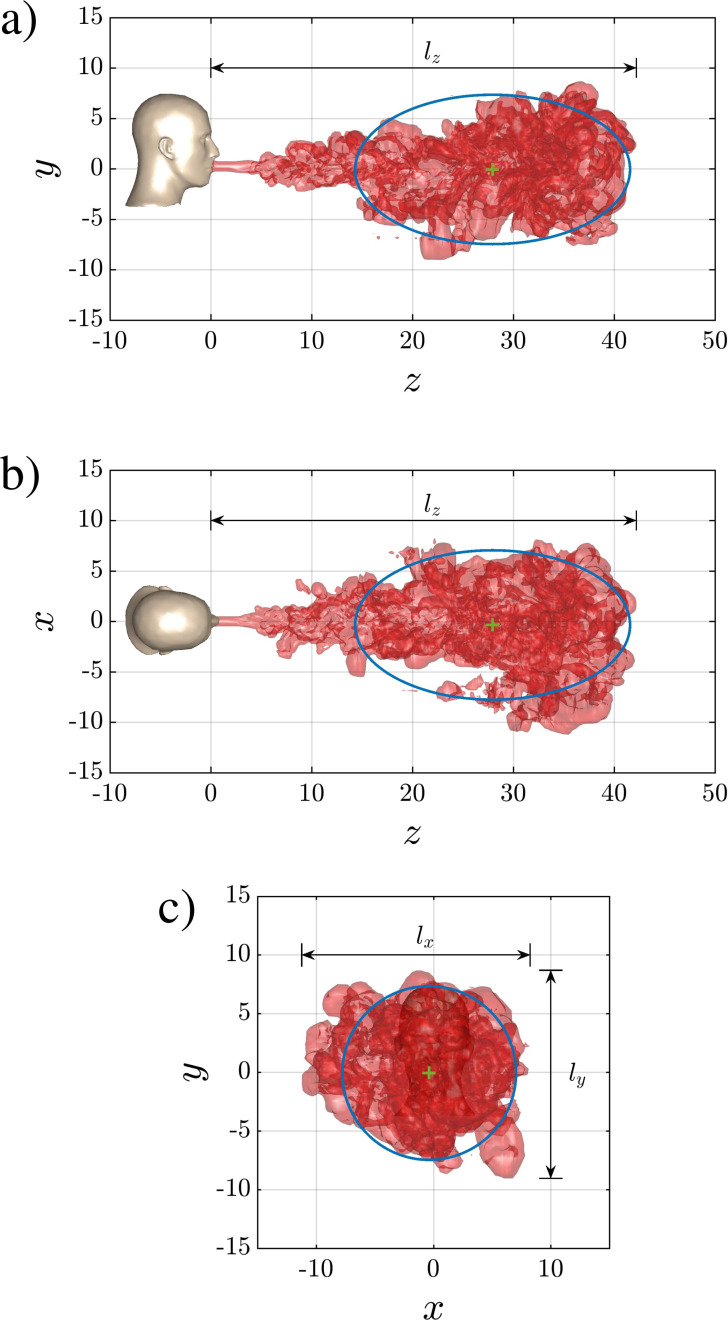
Projections of the turbulent puff from Fig. 2 at *t *=* *728 for Case Q10V20d along the (a) *y*–*z*, (b) *x*–*z*, and (c) *x*–*y* planes. The blue ellipses and circle in panels a, b, and c correspond to the projection of a prolate spheroid, centered at the puff centroid (green + symbol), onto the respective plane. The streamwise and transverse lengths of the puff are denoted by *l_z_*, *l_x_*, and *l_y_* and define the maximum extent of the puff.

Figure 4 shows projections of the turbulent three-dimensional puff shown in Fig. 2 onto the *y*–*z*, *x*–*z*, and *x*–*y* planes. The projections represent the maximum extent of the puff in each direction. Due to entrainment, the puff expands in the transverse *x* and *y* directions, but the transverse extent of the puff remains smaller than the streamwise extent. During the time period shown in the figures, the buoyancy effect of the temperature difference between the puff and the ambient has been quite small. The statistical shape of the puff in the vertical *y*–*z*-plane is quite similar to that along the horizontal *x*–*z*-plane. As a result, the puff appears to take the shape of a prolate spheroid with the major axis oriented along the ejection direction.

Since the mouth's cross section is taken to be circular, and the ejection direction does not vary with time (i.e., the head remains fixed during the ejection process), the puff is statistically axisymmetric. This implies that the *y*–*z* and *x*–*z* projections are statistically identical in the absence of buoyancy effects, but the neglect of buoyancy effects is a valid assumption only at short times after ejection when the puff velocity and the turbulent fluctuations are significant. It should be noted here that if a non-axisymmetric, for example an elliptical, mouth cross section is chosen, then the spreading will be dependent on the initial non-axisymmetric cross section. Furthermore, the *x*–*z* and *y*–*z* projections of the puff will no longer be statistically equivalent, especially for relatively high ejection aspect ratios. It is well known that elliptical jets exhibit different rates of entrainment in the two transverse directions due to differing rates of shear thickening along the initial minor and major axes of the ellipse.^44,45^ In fact, such non-canonical spreading is also present in buoyancy driven flows such as thermals^46^ and gravity currents,^47–49^ albeit due to different mechanisms.

**FIG. 5.**
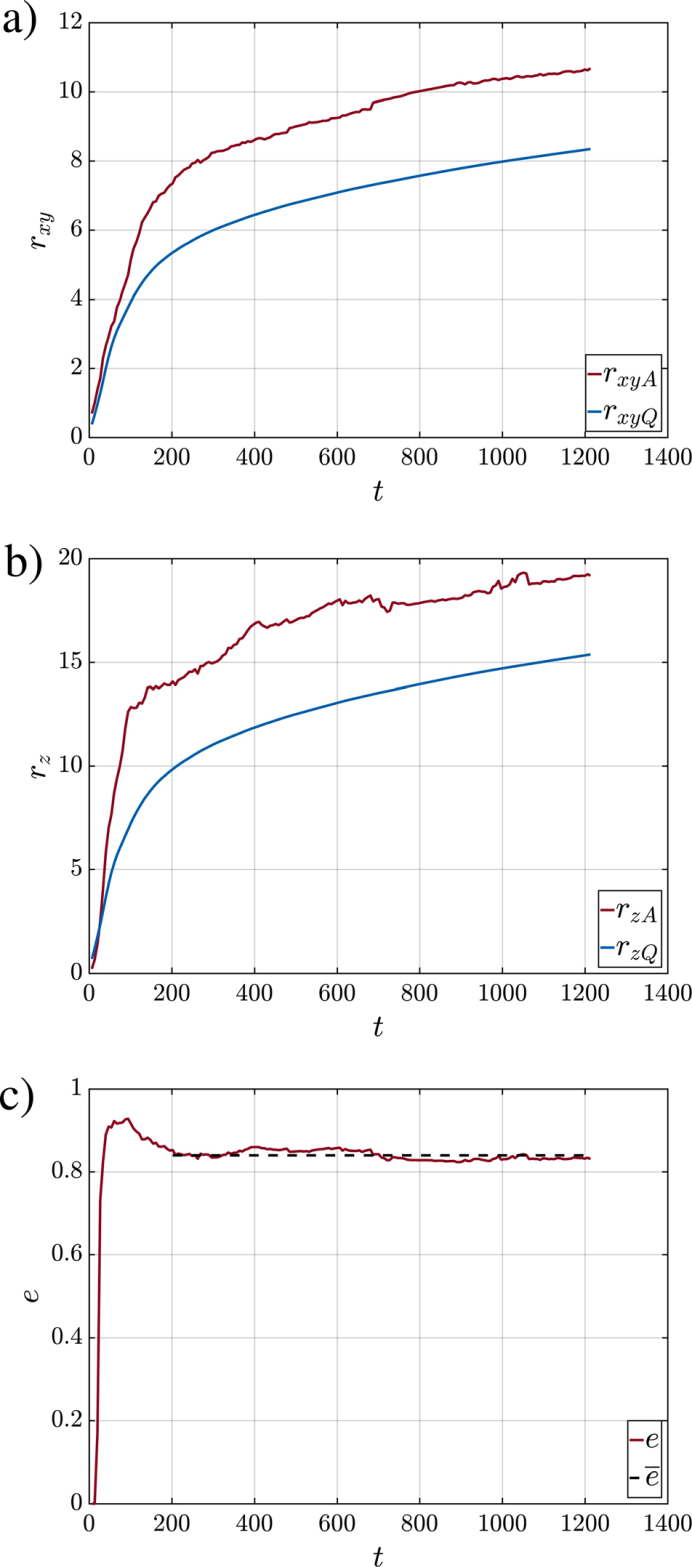
Time variation of (a) *r_xy_*, (b) *r_z_*, and (c) *e* for case Q10V20d.

Even though the shape of the puff is complex, showing surface undulations and large scale variations, for the sake of simplicity, the shape may be taken to be a prolate spheroid whose center corresponds to the puff's center. The semi-major axis *r_z_* and semi-minor axis *r_xy_* can be determined in terms of the projected areas as

$$r_{\mathrm{xyA}}=\sqrt{\frac{A_{z}}{\pi }}\;\text{and}\;r_{zA}=\frac{A_{x}+A_{y}}{2\pi \;r_{\mathrm{xyA}}},$$ (25)

where *A_x_*, *A_y_*, and *A_z_* are projected areas along these respective directions. The eccentricity of the spheroid then becomes $e=\sqrt{1-{\left(r_{\mathrm{xyA}}/r_{zA}\right)}^{2}}$. The time variation of *r_xyA_*, *r_zA_*, and *e* for Case Q10V20d are shown in Fig. 5 as the solid red curves in panels a, b, and c, respectively. The semi-major and minor axes increase monotonically over time except for small turbulent fluctuations. During the early portion of the ejection phase ( t≲130), where the majority of the ejected fluid is forced into the domain, the increase is relatively sharp. The rate of increase then slows down, but till *t *=* *271 the injection continues and contributes to increase in the projected puff radii *r_xyA_* and *r_zA_*. At later times, their increase is due to turbulent mixing and entrainment. Furthermore, beyond the ejection phase, we find the eccentricity to remain nearly constant at around 0.85 as indicated by the dashed black line.

### B. Evolution of global quantities

While the complex shape of the puff differs from one realization to another due to the amplification of small perturbations that were included to the otherwise identical ejection profiles, integral quantities such as the puff volume *Q*(*t*), momentum magnitude |M|(t), and buoyancy *B*(*t*) exhibit somewhat smaller variation. To evaluate the integral properties of the puff, an indicator function *I* is first defined based on a temperature threshold $T_{f,th}$ that separates the puff from the ambient as

$$I\left(x,y,z,t\right)=\{\begin{array}{cc} 1 & \text{if}\;T_{f}\geq T_{f,th}\\ 0 & \text{if}\;T_{f}<T_{f,th}\;. \end{array}$$ (26)

Several threshold values were tested. The results to be presented are for $T_{f,th}=0.002$ and the conclusions to be drawn have been verified to be independent of the particular value of this threshold. Once the puff is identified, relevant properties such as volume, momentum, and buoyancy can be easily obtained as

$$Q(t)={∭}_{\Omega }I\left(x,y,z,t\right)d\mathrm{xdydz},$$ (27)

$$M(t)={∭}_{\Omega }u_{f}I(x,y,z,t)\mathrm{dxdydz},$$ (28)

$$B(t)={∭}_{\Omega }T_{f}I(x,y,z,t)\mathrm{dxdydz},$$ (29)

where Ω represents the entire computational domain.

**FIG. 6.**
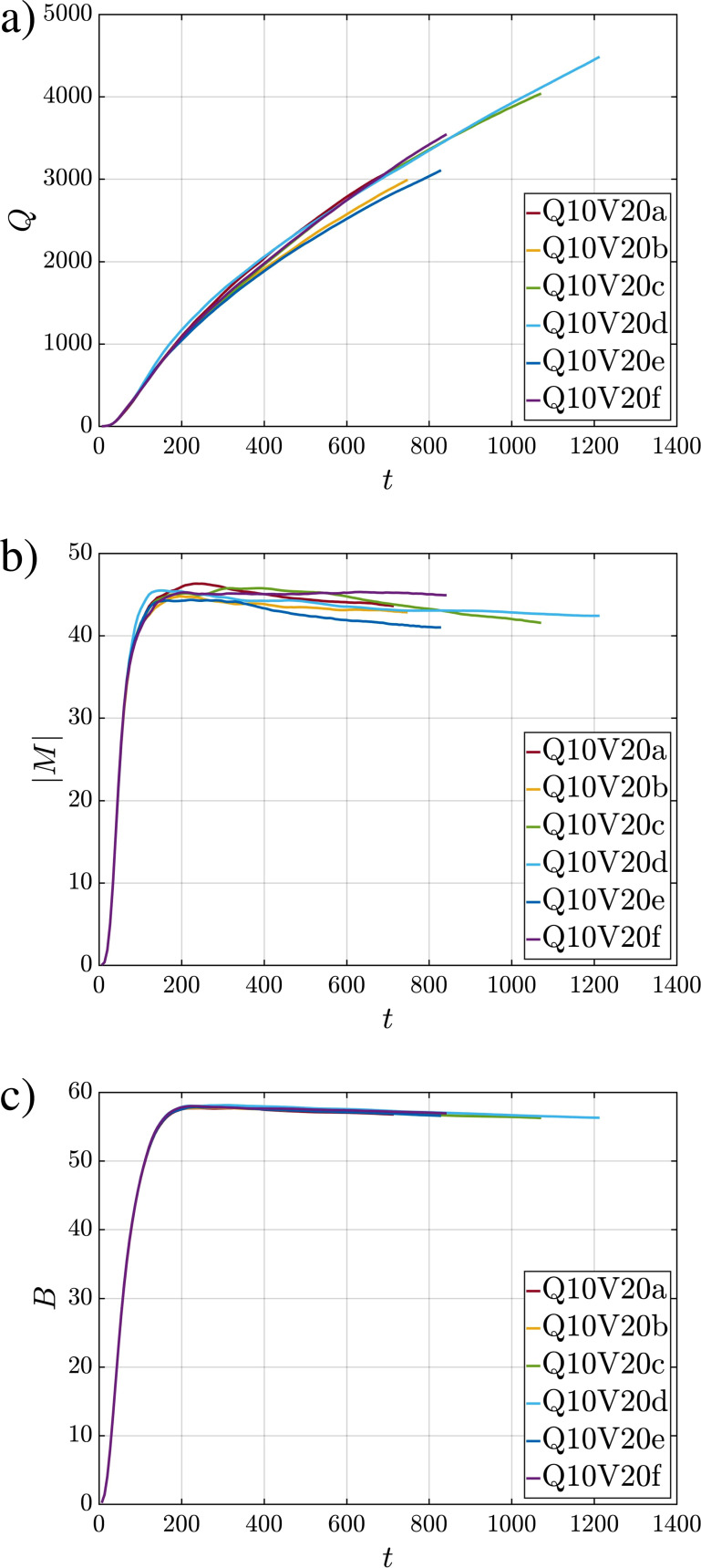
Temporal evolution of puff (a) volume *Q* [Eq. (27)], (b) momentum *M* [Eq. (28)], and (c) buoyancy *B* [Eq. (29)] for the various realizations from case Q10V20.

Figure 6 shows the temporal evolution of *Q*, |M|, and *B* for all simulations. The curves highlight the variability from realization to realization. This variability is larger in the case of volume and momentum compared to buoyancy, where all curves practically fall on top of one another. The initial increase in all three quantities is due to the continued injection of the fluid at the inlet. The injection ends at around *t *=* *271; however, the majority of the puff and droplets are injected by *t *=* *200 as seen in the injection profile of Fig. 1, which contains a relatively long tail. Beyond that time, a small portion of the puff and droplets are injected with low momentum. In the case of *Q*(*t*), the increase beyond the injection phase, which occurs at a slower rate compared to the initial injection phase, is due to the entrainment of ambient fluid into the puff. The precise measure of puff volume can be used to establish the size of the puff. Assuming the puff to be a prolate spheroid of eccentricity *e*, the semi-major and semi-minor axes of the puff based on the volume can be evaluated as

$$r_{zQ}={\left(\frac{3Q}{4\pi \;(1-e^{2})}\right)}^{1/3}\;\text{and}\;r_{\mathrm{xyQ}}={\left(\frac{3Q\sqrt{1-e^{2}}}{4\pi }\right)}^{1/3},$$ (30)

where the *Q* in the subscript indicates the semi-major and semi-minor axes evaluated based on puff volume, as opposed to projected areas. Time variations of *r_zQ_* and *r_xyQ_* are also plotted in Fig. 5, and they are substantially smaller than *r_zA_* and *r_xyA_*, respectively. The difference is primarily because the projections overestimate the actual cross-sectional area of the puff. Here, we use the projections to estimate the overall shape of the prolate spheroidal geometry in terms of the eccentricity and use that in conjunction with the volume of the puff to evaluate the volumetric semi-major and semi-minor axes. In Fig. 4, the projections of the prolate spheroid are also plotted on the *x*–*y*, *x*–*z*, and *y*–*z* planes as a blue circle of radius *r_xyQ_* and blue ellipses of semi-major and semi-minor axes *r_zQ_* and *r_xyQ_*.

Here, we have chosen a thermal threshold value of $T_{f,th}=0.002$ to define the boundary of the puff, since temperature serves as a marker that distinguishes the hot ejected fluid from the colder ambient fluid. The actual value of *Q*(*t*) and other quantities presented in Fig. 6 will depend on the precise value of $T_{f,th}$. At a larger (or lower) value of threshold the puff will become smaller (or larger), as can be expected. Although this means that quantities such as *Q*(*t*) somewhat depend on the threshold definition, the more important scaling parameters to be subsequently defined have been verified to be insensitive to the precise choice of $T_{f,th}$.

After the initial rapid increase during the ejection period, the momentum of the puff decreases, which provides evidence of the frictional resistance to the forward motion of the puff due to drag against the ambient fluid. As discussed in the theoretical model of Balachandar *et al.*,^1^ the velocity of the puff decreases due to both entrainment and ambient drag. In contrast, the total momentum of the puff is unaffected by the entrainment process and thus the decrease in momentum is entirely due to drag. At even later times than what is considered in Fig. 6, when the velocity of the puff has sufficiently fallen down, the effect of buoyancy can contribute to an increased vertical momentum of the puff.^50^ In the present context, this mechanism is always active, but at early times, however, the puff remains momentum-dominated and the effect of buoyancy is weak.

The total buoyancy of the puff *B*(*t*) increases again during the injection period. It is expected to reach its peak at the end of injection and maintain its value thereafter. This expected behavior is observed in Fig. 6. While total buoyancy is conserved according to the governing equations, the slow decay of *B*(*t*) in Fig. 6 is due to the fixed non-zero thermal threshold. Over time, some of the thermal energy of the injected fluid diffuses beyond the puff boundary (defined by the threshold) into the ambient, resulting in the slow reduction of *B*(*t*). When buoyancy (or temperature) is integrated over the entire volume of the computational domain, we observe *B*(*t*) to be strictly conserved verifying the accuracy of the numerical methodology employed in the present simulations. Finally, we note that the effect of buoyancy can be seen in Fig. 2 where the puff in the quiescent near-mouth region is observed to continuously rise between panels a, b, and c.

The volumetric center of the puff is defined as

$$x_{c}=\frac{1}{Q}{∭}_{\Omega }\left(xI\right)d\mathrm{xdydz}\;.$$ (31)

**FIG. 7.**
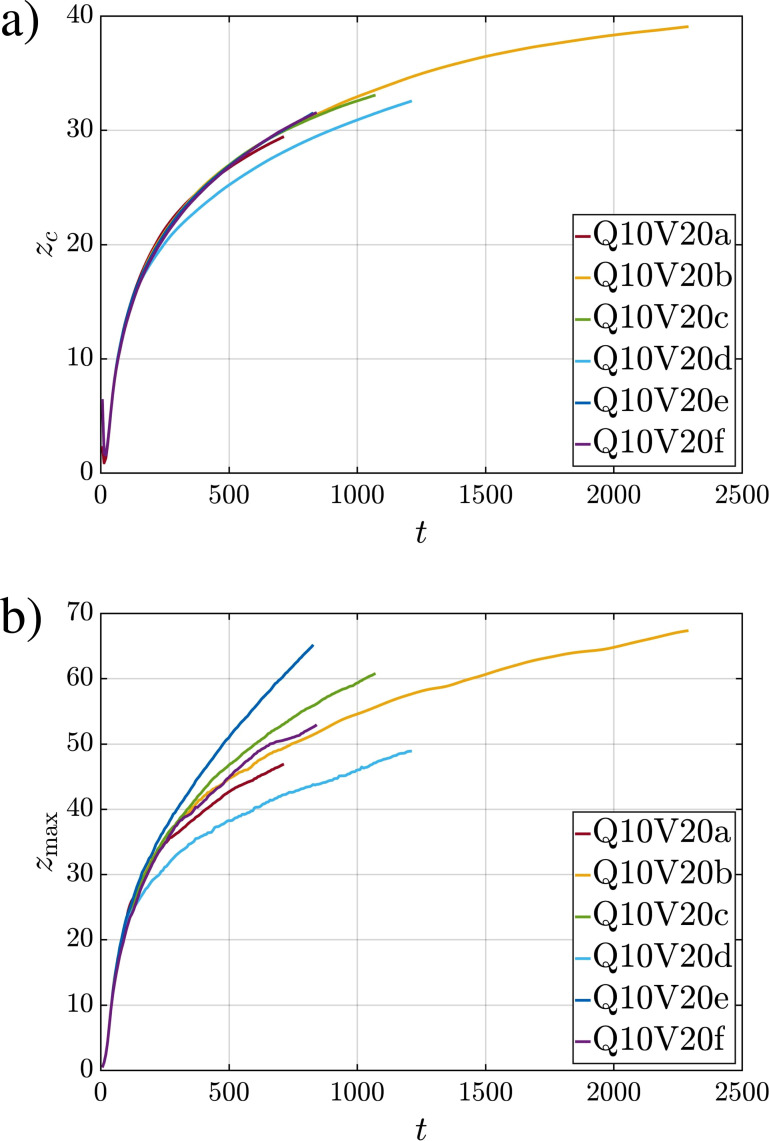
Temporal evolution for all simulations of (a) the puff volumetric center and (b) the maximum extent along the flow direction.

Figure 7(a) shows the time evolution of the *z* component of the volumetric center of the puff as a function of time for all cases. The transverse components of the center position, *x_c_* and *y_c_*, remain much smaller compared to the streamwise component *z_c_*. The ensemble average of *x_c_* obtained by averaging over many realizations is expected to be zero, since there is no mean ejection or net force along the *x*-direction. On the other hand, the *y*-component will statistically increase over time due to buoyancy. For all cases considered, we find the transverse components of the puff center location to remain one or more orders of magnitude smaller than the streamwise component, indicating that buoyancy is still not globally important up to five or more ejection times. We should note, however, that buoyancy may be relevant locally in regions of low turbulent intensity such as the near mouth region. Nonetheless, these quiescent regions only constitute a small fraction of the puff.

Figure 7(b) shows the time evolution of the maximum extent of the puff along the flow direction for all cases considered. This is a quantity of interest since it measures the farthest distance reached by the puff and the droplets within at any given time. As expected, the realization-to-realization variation in the volumetric center of the puff is much smaller than the corresponding variation in the farthest downstream extent of the puff. Even in the case of the volumetric center, the result from Q10V20d is lower than those of the other five cases mainly because the puff remains as one coherent body. Thus, random variations in the individual realizations have a modest effect on statistically averaged quantities [an effect that was observed earlier in the plots of *Q*(*t*) in Fig. 6]. These differences are however greatly amplified in the case of extreme statistics, such as the farthest streamwise extent of the puff. The detaching pockets allow the puff to extend farther in the streamwise direction. For example, at *t *=* *800, the puff extends up to $z_{\text{max}}\approx 64$ in Case Q10V20e compared to $z_{\text{max}}\approx 43$ in Case Q10V20d, for which the puff remains coherent.

### C. Scaling relation

We now evaluate the scaling relation of the puff location presented in Balachandar *et al.*,^1^ for which the streamwise location of the puff center is given by the power-law,

$$\frac{z\prime_{c}(t\prime )+z_{vo}}{z_{vo}}={\left(\frac{t\prime +t_{vo}}{t_{vo}}\right)}^{\frac{1}{4+C}},$$ (32)

where $z\prime_{c}$ is the distance traveled by the puff from the time t′ of its injection. Definitions for t′ and $z\prime_{c}$ are given in Eq. (34). According to the theoretical formulation, the puff of volume *Q_e_* was fully formed at time t′=0, whereas in the simulations, the injection process extended from *t *=* *0 to *t* = *t_e_*. Although the injection process was complete only at *t_e_*, the bulk of the injection happened at an earlier time when the injection velocity was at its peak (see inset of Fig. 1). An objective definition of the timescale of the injection process is

$$t_{\mathrm{inj}}=\frac{{\left(\int_{0}^{t_{e}}u_{e}(t)\;dt\right)}^{2}}{\int_{0}^{t_{e}}u_{e}^{2}(t)\;dt}\;\text{and}\;z_{\mathrm{inj}}=z(t=t_{\mathrm{inj}}),$$ (33)

where the puff location at the injection time is taken to be *z_inj_*. With this definition, the distance traveled by the puff and time elapsed from injection are given by

$$t\prime =t-t_{\mathrm{inj}}\;\text{and}\;z\prime (t\prime )=z(t)-z_{\mathrm{inj}}.$$ (34)

In Eq. (32), *t_vo_* and *z_vo_* represent the time and location of the virtual origin of the puff from the time and location of injection (i.e., from t′=0 and z′=0). In the power-law exponent, *C* is a constant that depends on the drag coefficient. Assuming the puff to be spherical, Balachandar *et al.*^1^ estimated the upper bound of *C* to be *C *=* *0.375. As will be discussed in Sec. IV E, the drag exponent *C* of each simulation can be evaluated and the results are presented in Table III.

**TABLE III.** Coefficients of the scaling relation determining the puff location.

Simulation	*t_inj_*	*z_inj_*	*C*	*t_vo_*	*z_vo_*	*α*	*t_voT_*	*z_voT_*	*C_D_*
Q10V20a	116.09	14.74	0.095	42.46	16.63	0.27	2.45	10.02	0.057
Q10V20b	116.09	14.60	1.529	57.57	19.58	0.24	2.69	11.25	0.109
Q10V20c	116.09	14.51	0.327	51.56	19.17	0.26	2.47	10.69	0.198
Q10V20d	116.09	14.58	0.117	66.62	17.99	0.27	2.45	10.07	0.074
Q10V20e	116.09	14.70	0.132	67.96	20.85	0.24	2.79	11.51	0.075
Q10V20f	116.09	14.31	0.210	69.57	22.17	0.25	2.59	10.89	0.119

**FIG. 8.**
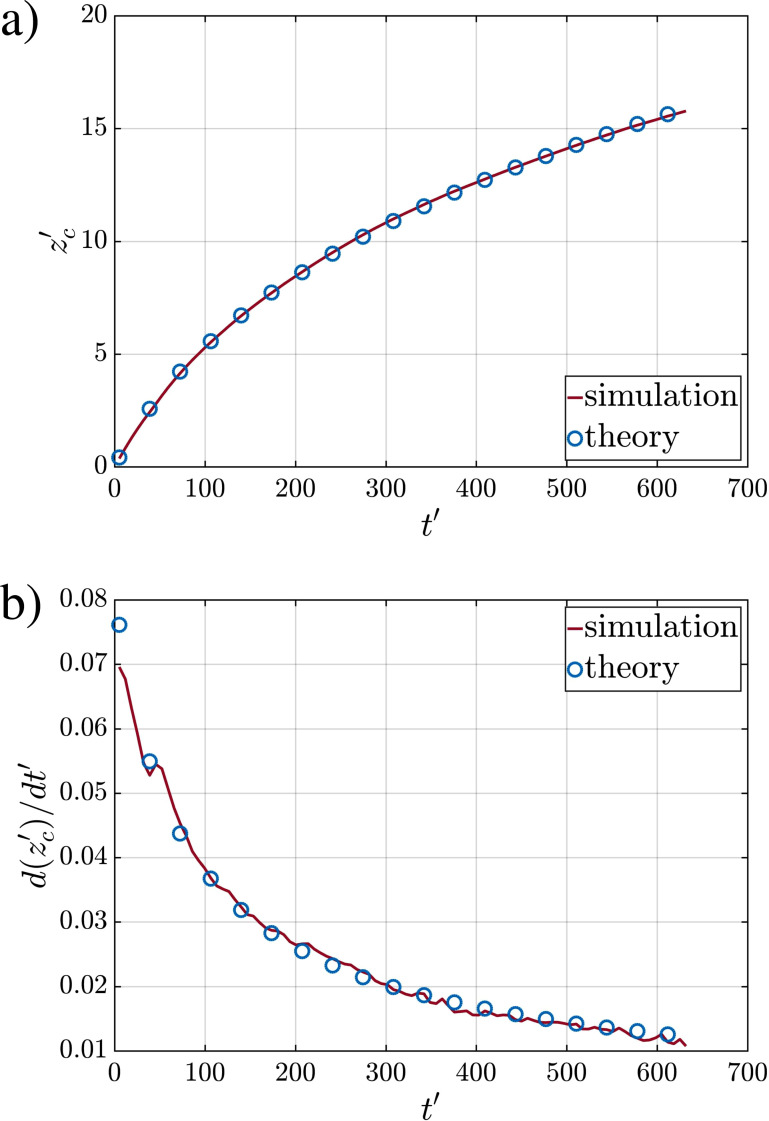
Plots of the puff volumetric center and its time rate of change. Temporal evolution of (a) puff volumetric center and (b) the corresponding time rate of change (i.e., velocity) for case-Q10V20b. The power-law solution of Ref. 1 (blue circles) shows excellent agreement with simulations.

The distance traveled by the puff z′(t′) in simulation Q10V20b is plotted in Fig. 8(a). Also shown in the figure, with circular symbols, is the best curve fit of the form given in (32). The agreement between the simulation results and the theory is quite good. The best fit is obtained with *z_vo_* = 19.58 and *t_vo_* = 57.57. According to the theory of Balachandar *et al.*,^1^ the virtual origin can be estimated as

$$\{\begin{array}{c} z_{\mathrm{voT}}=\frac{1}{\alpha L_{e*}}{\left(\frac{Q_{e*}}{\eta }\right)}^{1/3}\\ t_{\mathrm{voT}}=\frac{u_{e*}}{L_{e*}}\frac{Q_{e*}^{4/3}}{(4+C)\;\alpha \;M_{e*}\eta^{1/3}}, \end{array}$$ (35)

where the constant *η* is defined in terms of the effective puff radius *r_Q_* as $\eta =Q(t)/r_{Q}^{3}(t)$. If we take the effective radius of the spheroid to be $r_{Q}=\sqrt[{3}]{r_{zQ}r_{\mathrm{xyQ}}^{2}}$, then η=4π/3, which is the same for a spherical geometry. In the above, *α* is the entrainment coefficient, which we shall discuss in detail in Sec. IV D, and its value for the different simulations is listed in Table III. Using these values we obtain *z_voT_* = 11.25 and *t_voT_* = 2.69 for Case Q10V20b, which is compared with the values obtained from the best fit in Table III. Clearly, the theoretical scaling given in (35) must be appropriately scaled to predict the true virtual origin.

Figure 8(b) shows the *z*-velocity of the puff evaluated based on the volumetric center. The new power exponent obtained from differentiating (32) now becomes −(3+C)/(4+C). Here also, the symbols correspond to the theoretical prediction, where the constant value of *C *=* *0.217 represents the average value of *C* from the six realizations shown in Table III. The simulation results are in excellent agreement with the theory.

### D. Entrainment

Entrainment is the process by which ambient fluid is incorporated into the puff and thereby the volume of the puff steadily increases as seen in Fig. 6. Following the pioneering work of Morton,^51^ the velocity at which the ambient fluid enters the puff is taken to be a fraction of the streamwise velocity of the puff, and the ratio between the two is defined as the entrainment coefficient. In the present context, the entrainment coefficient can be calculated in a few different ways, and the entrainment coefficient obtained from these different approaches is likely to vary slightly from one definition to another.

**FIG. 9.**
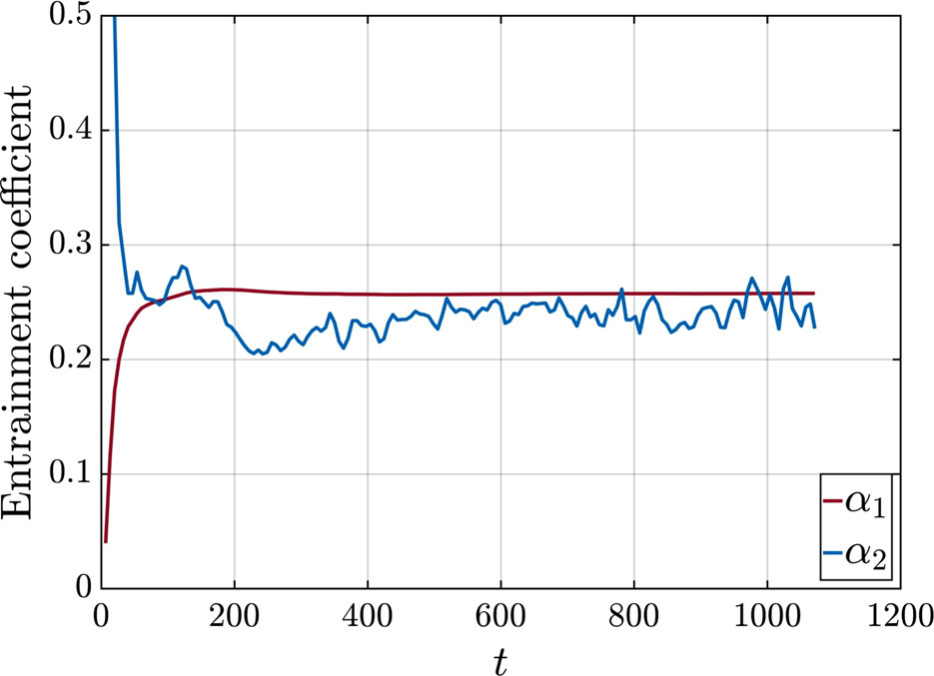
Entrainment coefficients *α*_1_ and *α*_2_ as a function of time for case Q10V20c.

Here, the following two definitions of entrainment are explored:

$$\alpha_{1}=\frac{r_{Q}(t)}{z\prime_{c}(t)+z_{vo}}\;\text{and}\;\alpha_{2}=\frac{dQ/dt}{A_{\mathrm{surf}}\;ds/dt},$$ (36)

where the first definition is based on the effective geometric cone etched by the puff evolution and follows the definition of Refs. 1 and 8. In the second definition, which is the more conventional definition, $(dQ/dt)/A_{\mathrm{surf}}$ corresponds to the velocity with which the ambient fluid enters the puff, and thus in this definition, *α*_2_ is defined as the velocity ratio. The surface area $A_{\mathrm{surf}}(t)$ is taken to be the surface area of the prolate spheroid of volume *Q*(*t*) and eccentricity *e*. Both entrainment coefficients, *α*_1_ and *α*_2_, are plotted as a function of time in Fig. 9. We find both coefficients to attain a near constant value beyond the ejection phase with $\alpha_{1}\approx 0.22$ and $\alpha_{2}\approx 0.25$. Both *α*_1_ and *α*_2_ are on the same order of the entrainment coefficients of plumes and thermals (e.g., Refs. 51–55).

### E. Frictional drag on the puff

**FIG. 10.**
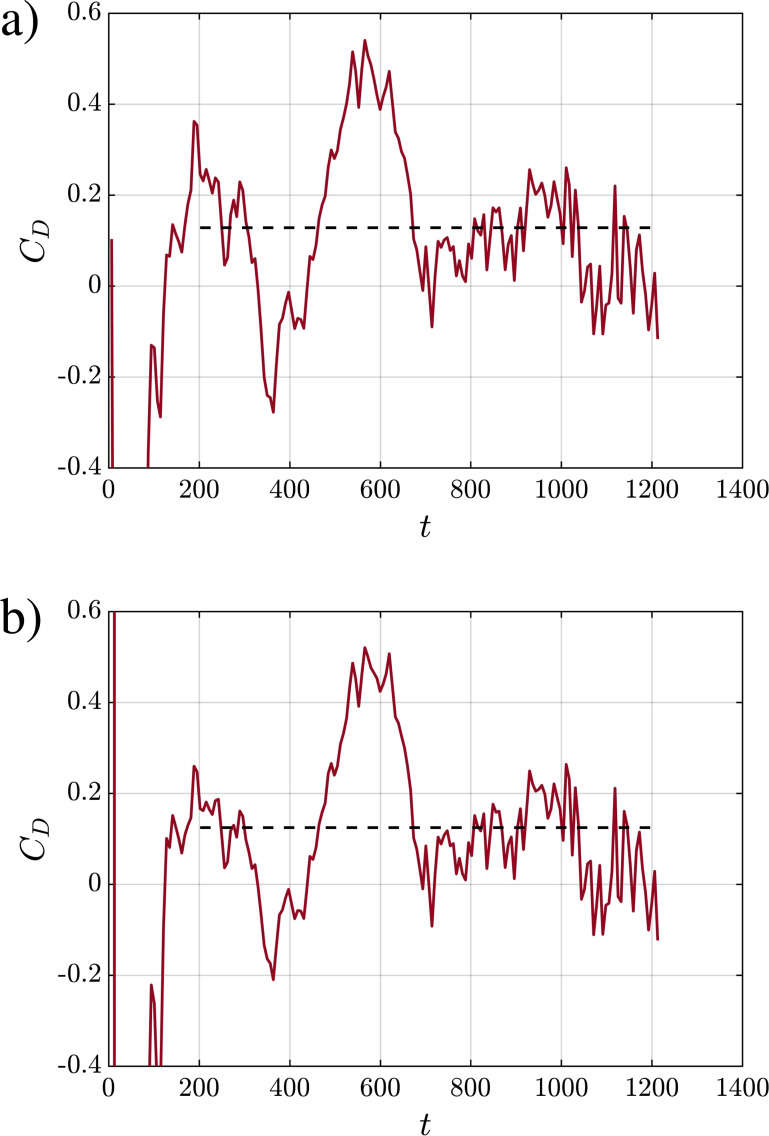
Drag coefficient *C_D_* vs time as defined by^1^ for Q10V20d based on (a) volume-weighted and (b) temperature weighted variables.

Another important empirical input to the theoretical framework that can be extracted from the simulations is the drag coefficient. Classical drag correlations will not be appropriate in the present case, since the puff can neither be treated as a solid sphere nor as a gas bubble with internal circulation. It cannot be treated as a porous sphere as well. Furthermore, the size of the puff shows strong variation over time. Following Balachandar *et al.*,^1^ the drag coefficient of the puff is defined as

$$C_{D}=\frac{dM_{z}/dt}{\frac{1}{2}\rho_{a}\pi r_{zQ}^{2}{\left(dz_{c}/dt\right)}^{2}},$$ (37)

where we have taken the area to be the projected area of the prolate spheroid of volume *Q*(*t*). The time history of *C_D_* is presented in Fig. 10 for volume-weighted (panel a) and temperature-weighted (panel b) variables. Since both approaches result in similar values, only the volume-weighted approach will be retained in the manuscript, unless stated otherwise.

According to the theory,^1^ the drag coefficient and the drag exponent in the power-law expression are related as follows:

$$C=\frac{C_{D}\beta }{2\eta \alpha },$$ (38)

where the constant $\beta =\pi r_{zQ}^{2}/r_{Q}^{2}$. The drag exponent computed as given above is presented in Table III. It must be pointed out that due to the large fluctuations seen in the time evolution of *C_D_*, its average value given in Table III must be interpreted as having a large error bar. Accordingly, the drag exponent given in Table III is likely to have large uncertainty as well. In the scaling relation (32), the drag exponent is added to 4; recall from (32) that the exponent is ${\left(4+C\right)}^{-1}$, and thus the curve fit is insensitive to large variations in the value of *C*. In other words, the scaling relation and the curve fits presented in Table III are somewhat insensitive to drag and can be computed ignoring the effect of drag.

## V. DROPLET EVOLUTION

### A. Size, spatial distribution, and trajectories

**FIG. 11.**
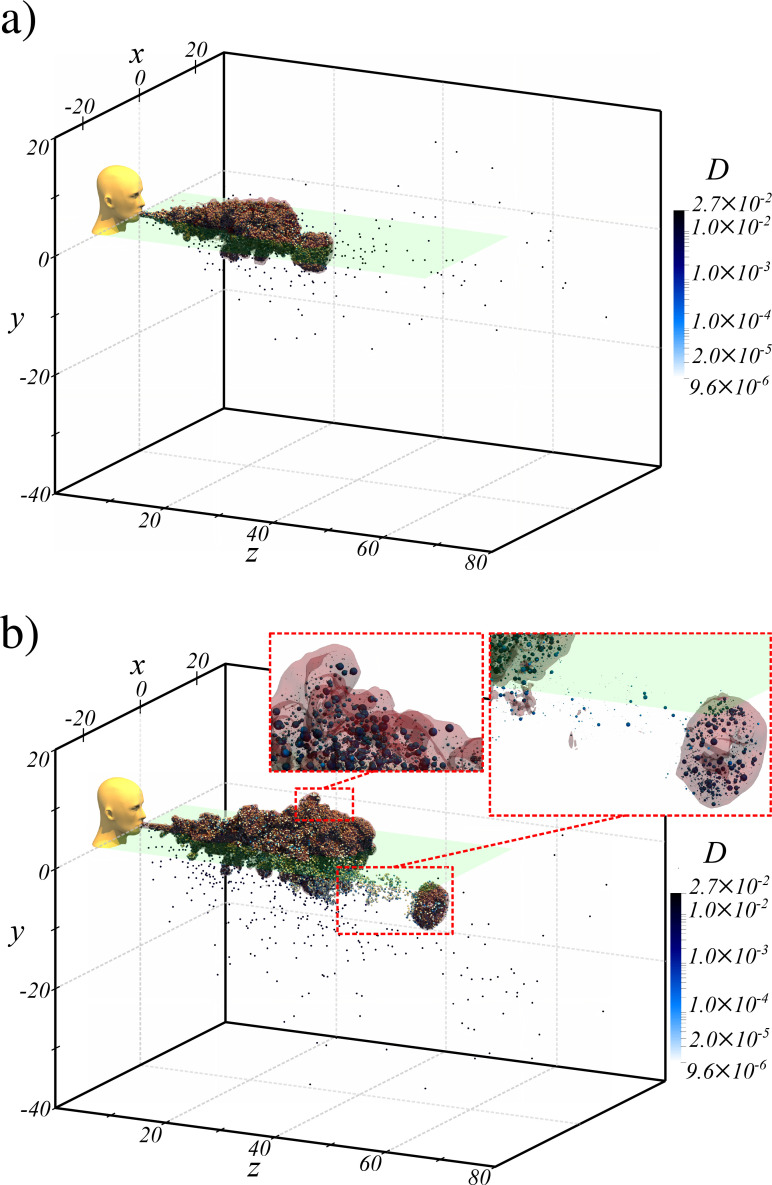
Isometric view of droplet distribution with respect to the puff shown as a semi-transparent iso-surface of $T_{f}=0.01$ for Q10V20e (a) immediately before separation of a small portion of the cloud and (b) after separation. All droplets are given the same size but are colored by droplet diameter. The insets in panel b show two magnified regions where the droplets relative size is maintained (relative to one another), but magnified by a factor of 500 for clarity.

In this section, we will consider Case Q10V20e as an example and examine the droplets size and spatial distribution as well as their trajectories with respect to the puff. In Fig. 11, we show an isometric view of the puff and the droplets. The puff is visualized with a semi-transparent iso-surface of $T_{f}=0.01$ and droplets are colored by diameter and are given a uniform size so that all droplets become visible, for otherwise the smaller droplets cannot be detected in the figure since the droplet diameter varies by three orders of magnitude.

The droplets and the puff are shown at two time instances, namely *t *=* *243 and *t *=* *728, in panels a and b, respectively. The *y *=* *0 plane is also shown as a semi-transparent surface to help gauge the vertical location of the puff and droplets. We find the majority of droplets to be contained within the puff as indicated by the relatively dense (in terms of droplet number density) droplet cloud that takes on the same shape as the puff. A small number of droplets do, however, overshoot or settle out of the puff. These are the largest droplets (as indicated by their dark color) that move near-ballistically.^1^ In contrast, the droplets that remain within the puff are small as indicated by their light surface color. When these droplets are just outside the puff, their true light color is visible; however, when they are within the puff, their color is influenced by the semi-transparent puff iso-surface and by the *y *=* *0 plane.

In panel a (respectively, panel b), a small portion of the puff is about to separate (respectively, has separated) from the main body of the puff. This portion advances at a faster speed than the puff and is able to drag along a proportionate number of droplets and keep them in suspension within. While the larger droplets are able to reach larger distances due to their near-ballistic motion, the puff separation provides another mechanism for transporting droplets, in larger numbers, to farther extents. A large number of droplets will therefore faithfully follow the peeled-off portion of the puff, while some droplets will become stranded between the main body and the separated portion. Because of the very large density difference between the droplets and the surrounding air, droplets will continuously settle out of the puff as shown in panel b. Panel b also shows two magnified views belonging to the main body of the puff as well as the separated portion. In these magnified views, the droplet's actual diameter is scaled up by a constant factor of 500 to render the nearly invisible micrometer-sized droplets visible. We observe a wide range of droplet size in each of the two magnified views.

**FIG. 12.**
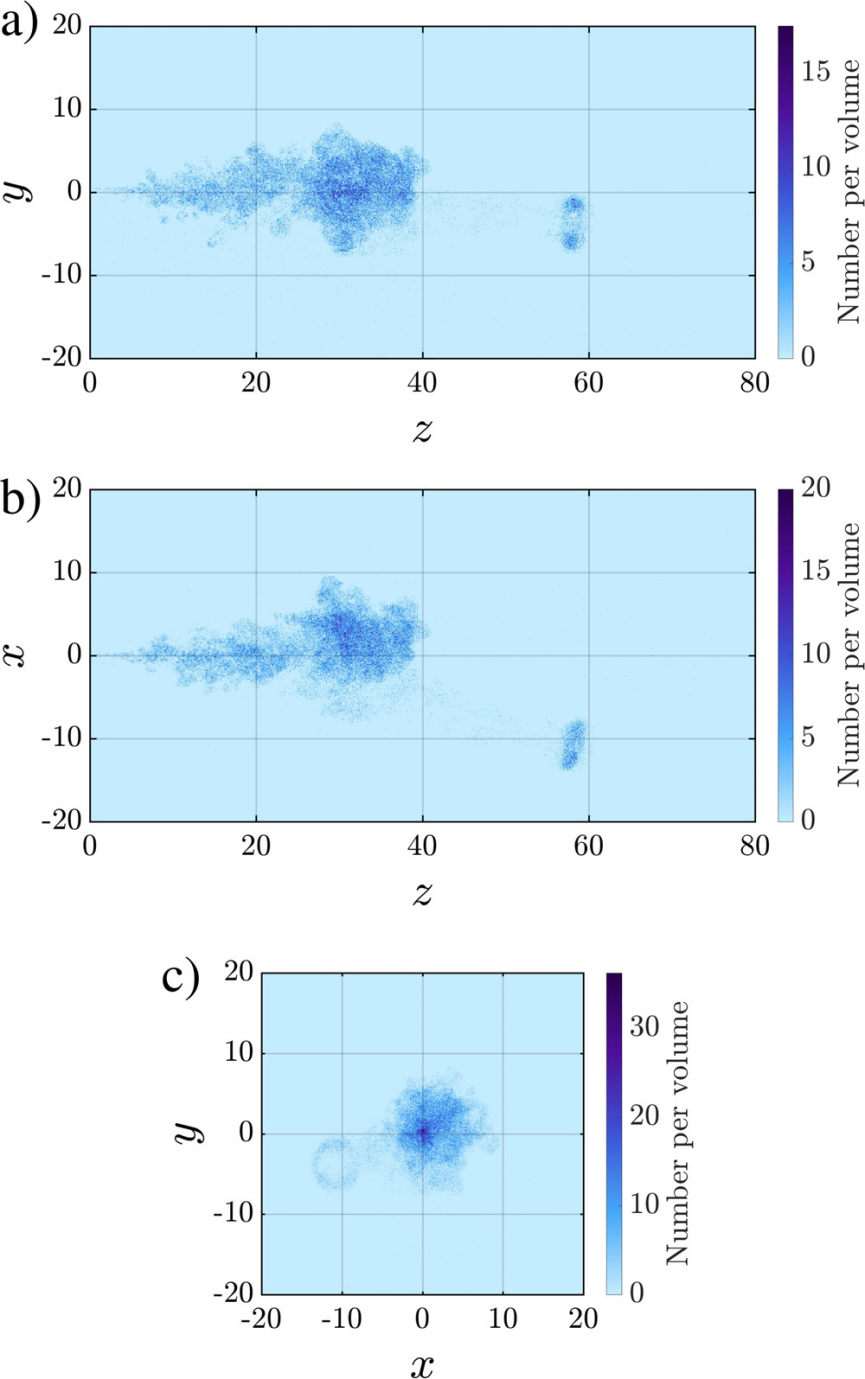
Projections of the droplet cloud from Case Q10V20e onto the (a) *y*–*z*, (b) *x*–*z*, and (c) *x*–*y* planes along with the droplet number density.

Figure 12 shows projections of the number density contours on the *y*–*z*, *x*–*z*, and *x*–*y* planes. The number density plots reflect the number of droplets per unit volume within a rectangular cuboid whose length corresponds to the length of the numerical domain along the projected direction, and whose two other dimensions correspond to a square of side length 0.1. The value of *n_d_* would thus represent the probability of finding a droplet in the respective rectangular cuboid multiplied by the ejected number of droplets. Under perfect axisymmetry, the highest number density can be expected to occur in a circular area around the puff axis (i.e., around the x=y=0 line). However, as seen from Fig. 12, the droplet cloud is not perfectly symmetric due to the turbulent nature of the flow. The location of the highest density of droplets is observed to vary from realization to realization.

We note that the ring-like shape of the separated portion of the puff can be inferred from the projected number density contour in the *x*–*y* plane where the center of the separated puff is practically void of any droplets. The path that the separated portion of the puff takes and its angle with respect to the main body may also be inferred from the *x*–*z* and *y*–*z* projections by tracing the droplets that are stranded between the main body and separated portion of the puff.

**FIG. 13.**
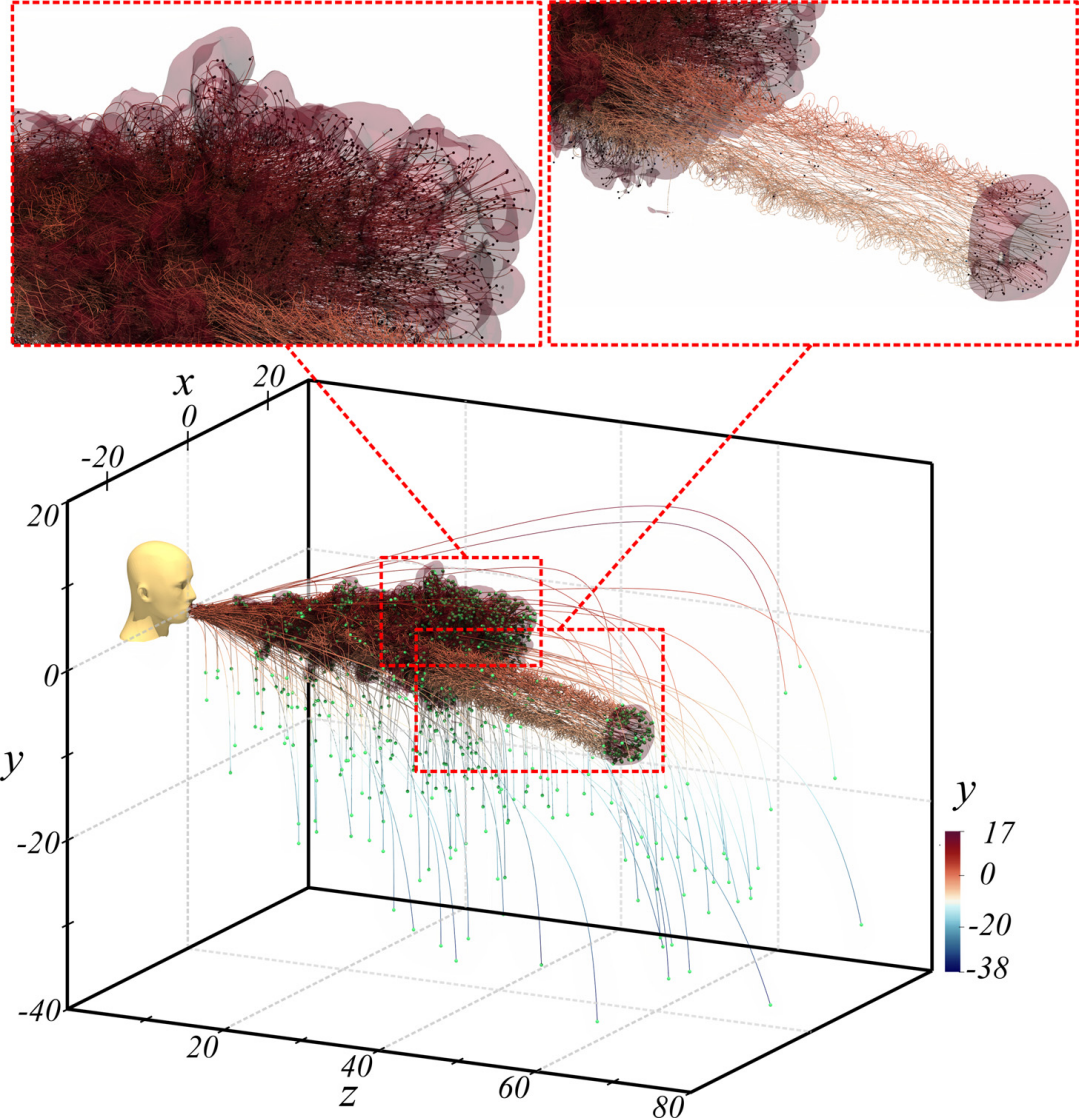
Droplet trajectories Q10V20e.

Since the Stokes number of the smaller droplets that remain within the puff is small, these droplets faithfully follow the fluid motion, and thus their trajectories may be used to infer the turbulent motion within the puff. Their settling motion with respect to the surrounding fluid cannot be discerned, since their still-fluid settling velocity is much smaller than the fluid velocity within the puff. The droplet trajectories for Case Q10V20e at *t *=* *829 are shown in Fig. 13. Here again, the puff is defined by the semi-transparent $T_{f}=0.01$ isocontour, and all droplets are shown with the same size for visibility and clarity as discussed previously. We identify two types of droplets: (i) relatively large droplets of near-ballistic trajectories^1^ whose motion is largely unaffected by the puff dynamics, but depend on the velocity at the time of their ejection; (ii) relatively small droplets whose complex three-dimensional trajectory is heavily affected by the surrounding puff velocity. For the near-ballistic motion, we clearly observe the influence of gravity where the trajectory becomes nearly aligned with the vertical *y* axis as the velocity of the droplet is reduced due to drag.

Figure 13 also shows two enlarged views in the main body of the puff as well as in the separated portion. The enlarged view for the main body shows the chaotic and complex motion of the dense droplet cloud within the puff, which is again an indication of the turbulent nature of the puff. On the other hand, for the separated portion of the puff, we observe a helical trajectory of particles that correspond to the vortex ring-like structure of the separated puff.^7,46^ The droplets contained within the separated puff are of various sizes as shown in the magnified view of Fig. 11(b).

**FIG. 14.**
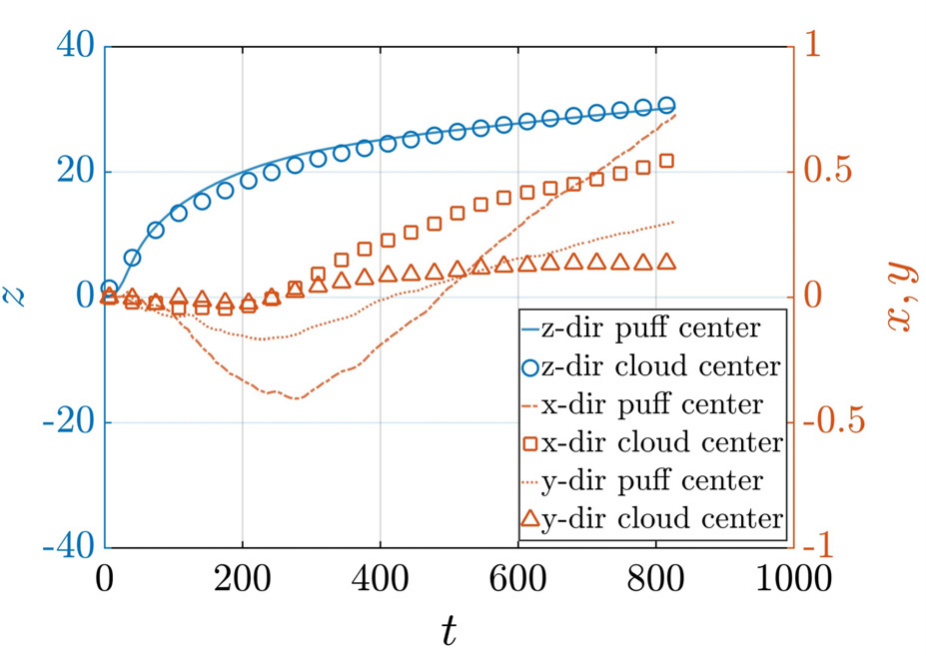
x, *y*, and *z* components of the volume-weighted center of the puff (dashed-dotted, dotted, and solid curves, respectively) and number-weighted center of the droplet cloud (squares, triangles, and circles, respectively) from Q10V20e.

**FIG. 15.**
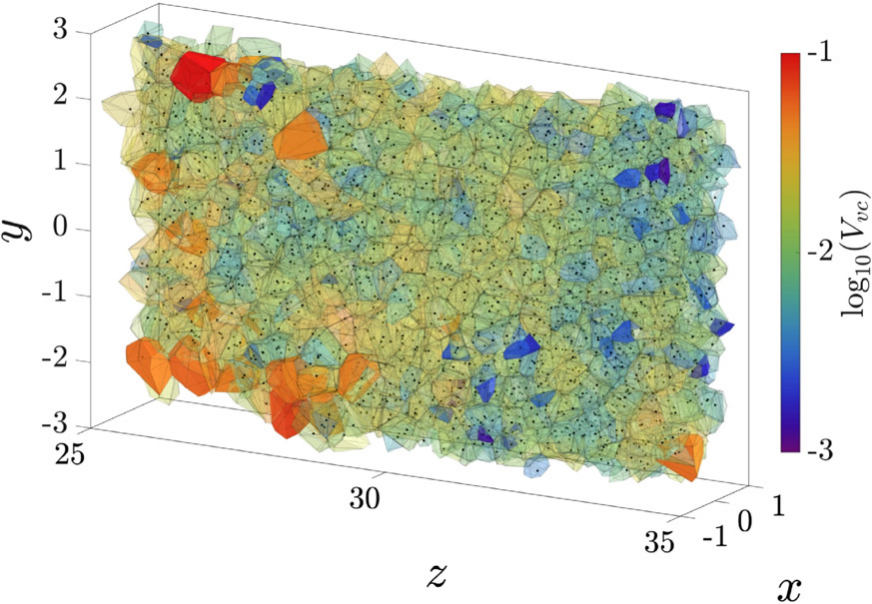
Voronoi tessellation of an inner region of the droplet cloud from case Q10V20e. The selected region within [−1,1]×[−3,3]×[25,35] is divided into a series of Voronoi cells colored by the cell volume. The clustered area contains smaller cells (blue), while the sparse area contains the larger (red). The mean cell volume is within [0.01,0.1].

Figure 14 shows the time evolution of the volume-weighted components of the center of the ejected puff of fluid along with the number-weighted components of the center of the droplet cloud, which is composed of the type-II droplets that stay within the puff. The fairly close agreement between the *x*, *y*, and *z*-components of the puff and droplet centers is indicative of the important fact that the evaporated droplet nuclei are well distributed within the puff. We should note that the relative difference between the *x* and *y* components of the centers appears to be large because both components fluctuate about zero. In reality, the absolute error is about the same for all three components. The agreement appears stronger for the *z*-component because it has a non-zero mean value. We also observe no stratification of the droplets within the puff due to gravitational settling. The near-uniform (unbiased) distribution of droplets within the puff only means that in a statistical sense the droplets are likely to be found in any part of the puff. There will be however preferential accumulation of droplets in certain regions of the puff as the small droplets are spun out of the turbulent vortices. That is, the uniformity of the droplet distribution is only true at the macroscale level, while at the mesoscale, the droplets will exhibit preferential accumulation. Figure 15 shows the Voronoi tessellation of an inner region of the droplet cloud. The blue color in the figure corresponds to regions with high droplet density, while the red color corresponds to regions with low droplet density. The figure thus demonstrates the non-uniform distribution at the mesoscale.

**FIG. 16.**
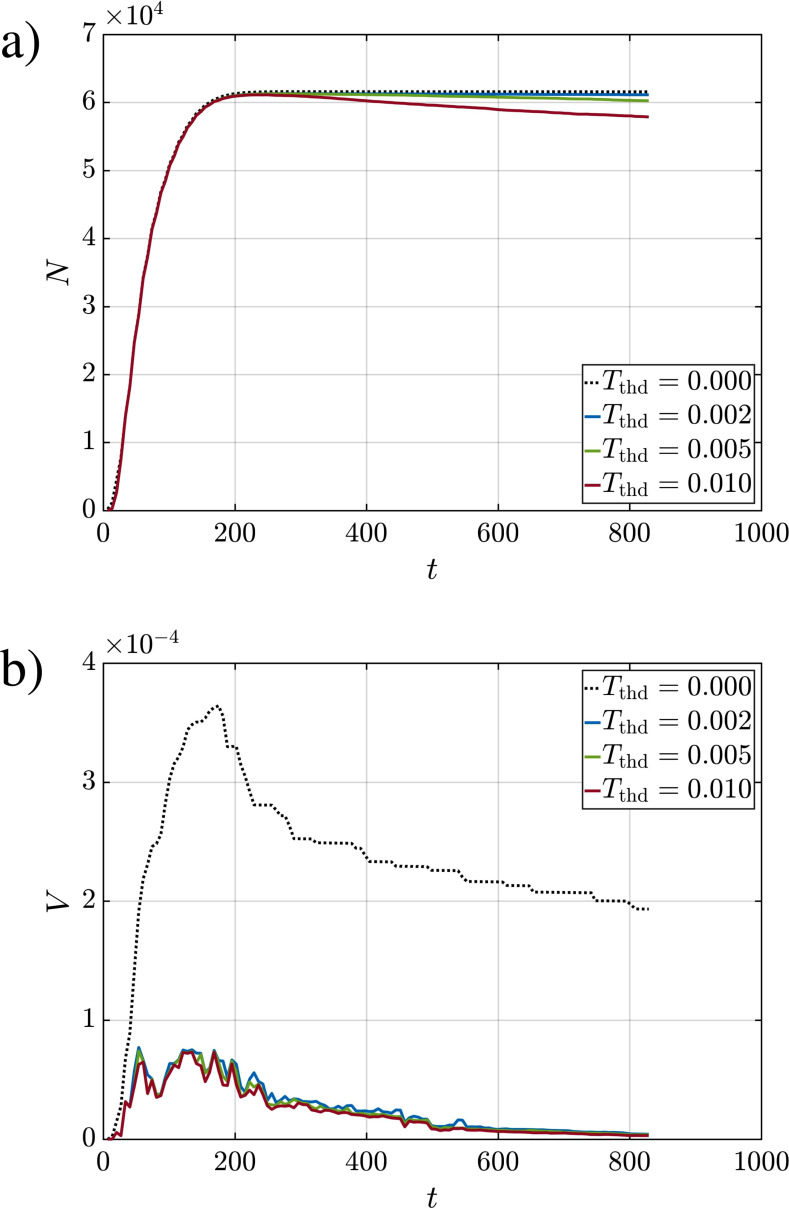
(a) Total number of droplets *N* from Q10V20e within the domain (dashed line) and within the puff (solid lines) for various temperature threshold values ( $T_{f,th}$). (b) Same as (a) but for total droplet volume.

### B. Global statistics

The droplets are ejected continuously during the ejection phase, and as such the number of droplets within the domain will increase steadily until the end of the ejection phase. As observed in Figs. 11–13, droplets are mostly contained within the puff. This observation is confirmed in Fig. 16, which again considers the simulation Q10V20e. In panel a, the number of droplets within the entire domain as a function of time is shown as the dashed black line. The number clearly increases until the end of the ejection phase and remains nearly steady at a value close to *N_e_* = 61650 thereafter. In reality, a few droplets exit the numerical domain due to their near-ballistic motion; however, these droplets constitute a very small fraction of the total number of ejected droplets. The number of droplets that remain within the domain is nearly unchanged beyond the ejection phase as seen in Fig. 16. The number of droplets within the puff, which is also shown in panel a for different temperature threshold values, further indicates that the majority of droplets remain within the puff. For example at *t *=* *800 and for $T_{f,th}=0.002$ (blue line), the number of droplets that remain within the puff constitute over 99% of the total ejected droplets.

**FIG. 17.**
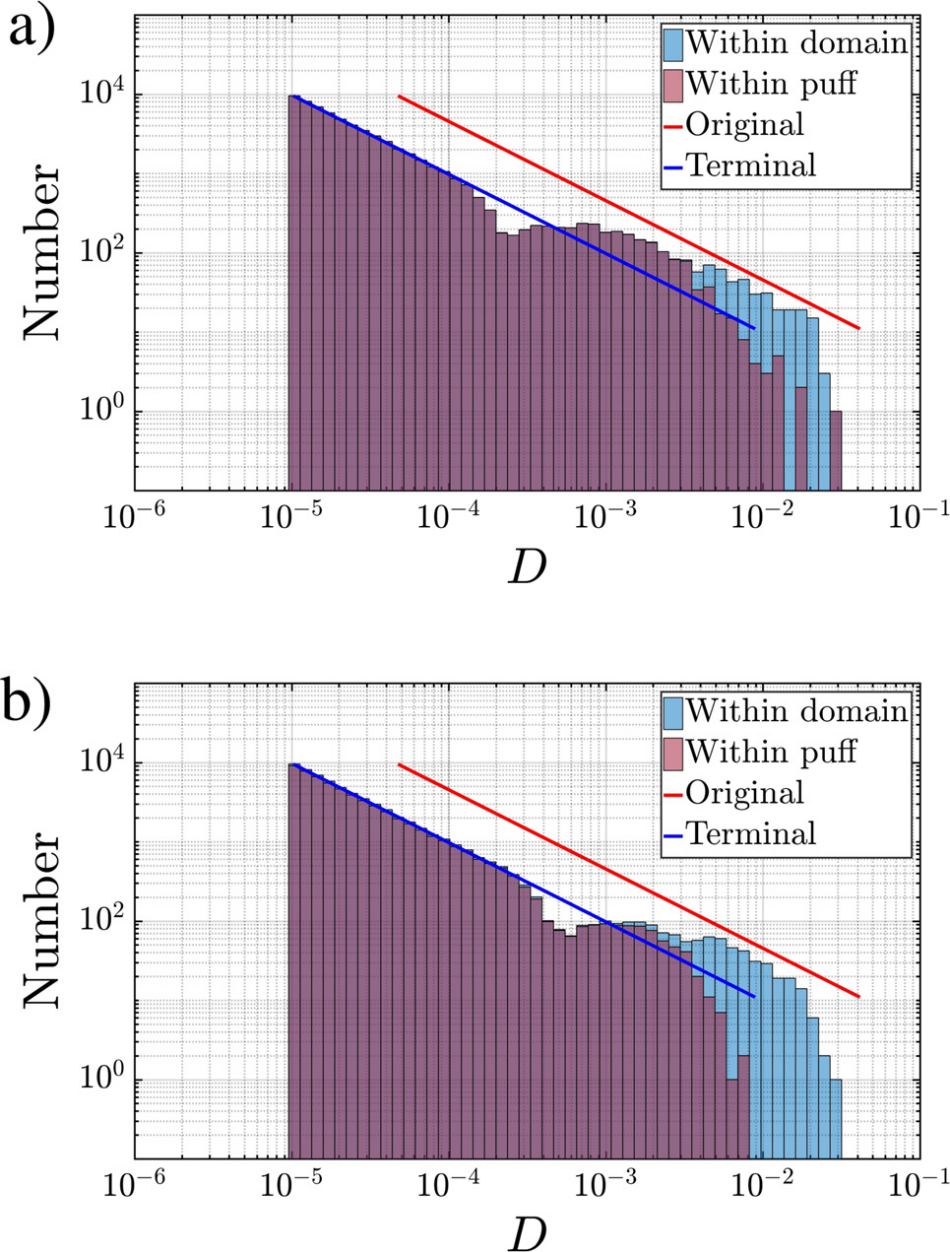
Droplet spectra from Q10V20e within the puff (purple) and within the domain (blue) at three time instances (a) *t *=* *243 and (b) *t *=* *728. The solid line corresponds to the injection droplet distribution profile.

While the number of droplets within the puff and the computational domain is nearly the same, the total volume of droplets contained within the puff is substantially lower than that within the domain as marked by the large difference in Fig. 16(b). We observe the dashed black line, which corresponds to the total volume of droplets within the computational domain, to be well above the total volume of droplets within the puff. This difference is due to the fact that the droplets that are outside the puff, though few in number, are much larger than those that remain within the puff.

The total volume of droplets within the domain increases sharply during the ejection phase up until t≊180 and then decreases continuously even during the ejection phase that ends only at *t *=* *271. The total volume decreases due to two factors. The first is due to droplets exiting the computational domain through any of the five outlet boundaries (top, bottom, left, right, and front). The second is due to evaporation as the droplet volume continuously decreases until the nonvolatile droplet core is reached, which in the present case is only 1% of the initial volume. Both these effects contribute to the reduction observed in Fig. 16(b). It is interesting to note that while the decrease in total droplet volume within the domain is monotonic beyond the ejection phase, the total droplet volume within the puff fluctuates over time. This is because one or more of the larger droplets may exit then occasionally reenter the puff at a later time due to the complex and turbulent dynamics of the puff.

The time evolution of the droplet size distribution within the puff as well as within the computational domain is influenced by the ejection droplet velocity and direction, the puff dynamics, as well as the droplet evaporation rate.^56–58^ The droplet size distribution from Case Q10V20e is shown in Fig. 17 at two time instances *t *=* *243 and *t *=* *728 in panels a and b, respectively. The initial ejection size distribution is marked by the dashed orange line and corresponds to the Pareto size distribution^1^ as mentioned previously. After complete evaporation, the size of the remaining droplet nuclei is 21.5% of the initial diameter (for the present 1% by volume nonvolatile). Thus, each droplet of size smaller than *D_exit_* that remains within the puff has reduced in diameter, which simply corresponds to a left-shift of the spectrum in the log –log plot. This final expected left-shifted spectrum is shown in the figure as the blue line.

Also shown in the figures are the droplet number histograms for all the droplets that remain within the computational domain (blue histogram) and those that remain within the puff (purple histogram). At *t *=* *243 only droplets of size $D<2\times 10^{-4}$ are fully evaporated and the histograms are in good agreement with the final expected spectra (blue line). By *t *=* *728 droplets of size $D<4\times 10^{-4}$ have fully evaporated to form the droplet nuclei. Thus, as time evolves the distinct dip seen in the spectra propagates to the right (i.e., to a larger and larger droplet size) so that the histogram eventually matches the blue line. The difference between the blue and the purple histograms corresponds to droplets that have exited the buff by either overshooting or dropping out due to gravity. The very good agreement between the theory and the simulation results is quite encouraging. The theory can thus be used to predict the behavior of the puff and the airborne droplet nuclei under a wide variety of conditions.^59^

## VI. CONCLUSION

We presented the results from six large eddy simulations of a puff laden with over 61 000 droplets resulting from a model cough or sneeze. Droplets are individually tracked using the point-particle Euler–Lagrange approach. As far as the puff is concerned, the six cases represent different realizations since the ejection velocity and temperature profiles are identical across all simulations except for a small 5% random perturbation. Global quantities are observed to vary little from one realization to the other, while local and instantaneous quantities may show large differences.

One of the interesting findings was the detachment of a small portion of the puff. The detached portion resembles a vortex ring-like structure and advances at a relatively fast speed along a direction that slightly deviates from the flow direction, but is unknown *a priori*. The detached puff can travel over relatively large distances and is observed to carry along a proportionate amount of suspended droplets. The detached portion provides a mechanism to extend the reach of the puff in an arbitrary direction over a short period of time. The liquid in the droplets is observed to evaporate quickly leaving behind nonvolatile nuclei cores that may contain viruses. The vast majority of these evaporated droplets (over 99%) remain afloat within the puff well after ejection. Due to their very small size, they may remain afloat due to ambient turbulence after the puff's initial momentum decays.

The first main set of conclusions of this study corresponds to the validation of some of the important assumptions made in the theoretical framework of Balachandar *et al.*^1^ (i) It was confirmed that buoyancy effects of the ejected puff are quite small in the early stages of ejection. Note that this is true for the present case of a violent ejection. For milder ejections, such as in the case of speaking and breathing, the buoyancy effect can be significant even at early times. The only noticeable effect of buoyancy was on the slow moving fluid ejected at the tail end of the cough or sneeze. (ii) The puff cannot be approximated as a spherical volume. It is better approximated by a prolate spheroid of aspect ratio of about 0.85. (iii) The droplets that remain airborne as fully evaporated droplet nuclei are sufficiently small that their velocity can be approximated as a simple sum of the local fluid velocity and the still fluid settling velocity. (iv) The droplet evaporation rate has been confirmed to follow an effective *d*^2^-law.^59^

The second main set of conclusions relates to the closure coefficients that are needed in completing the theoretical framework. (i) The entrainment coefficient of the ejected puff during its initial evolution was observed to be around 0.25, on the same order as other thermals. Two different definitions for the entrainment coefficient were explored and both yielded consistent results. Due to entrainment, the volume of fluid within the puff increased substantially during its early evolution. (ii) The drag coefficient *C_D_* of the puff was determined to be typically smaller than 0.1. Although there was considerable uncertainty in its evaluation, it is clear that the forward motion of the puff (the velocity of the puff center) was mostly influenced by the entrainment process and to a much lesser extent by drag. The (1/4) power-law for puff motion is reasonably accurate even without the drag correction. (iv) The virtual origin's location and time were extracted from the simulations, and their theoretical prediction given in (35)^1^ must be scaled up to match the simulation results.

The third main set of conclusions also pertains to the validation of the theory. (i) It is observed that the puff size and puff location are well predicted by the theory provided the correct virtual origins are used in their evaluation. (ii) The size of the largest droplet that remains airborne within the puff is well captured by the theory. (iii) The number density of droplet nuclei that remains suspended within the puff is well captured by the left-shifted spectrum just as predicted by the theory.

## Data Availability

The data that support the findings of this study are available from the corresponding author upon reasonable request.
